# Neural Response Reliability as a Marker of the Transition of Neural Codes along Auditory Pathways

**DOI:** 10.1002/advs.202508777

**Published:** 2025-09-18

**Authors:** Alexa Buck, Typhaine Dupont, Rupert Andrews Cavanagh, Olivier Postal, Jérôme Bourien, Jean‐Luc Puel, Nicolas Michalski, Boris Gourévitch

**Affiliations:** ^1^ Université Paris Cité Institut Pasteur AP‐HP INSERM CNRS Fondation Pour I'Audition Institut de I'Audition IHU reConnect Paris F‐75012 France; ^2^ Collège Doctoral Sorbonne Université Paris F‐75005 France; ^3^ Institute for Neurosciences of Montpellier (INM) INSERM University of Montpellier Montpellier F‐34091 France

**Keywords:** auditory system, awake mice, computational neuroscience, decoding, electrophysiology, neural code

## Abstract

The neural representations of acoustic features that differ in the location or timbre of the emitter elicit similar perceptions, suggesting the existence of a robust stimulus‐response function between complex sounds and the activity of neural populations at all stages of the auditory system. This hypothesis is tested by decoding a random sound stream, using spike trains from a biophysical model of the auditory nerve and from large‐scale recordings in the inferior colliculus, the auditory thalamus, and the auditory cortex of awake mice. At the level of individual neurons, the reliability of temporal and rate codes is found to decrease along the ascending auditory pathways. Rate coding is progressively favored with increasing independence of neuron frequency tuning. Firing in the auditory cortex is found to be synergistic, whereas that in subcortical areas is more redundant. Finally, combinatorial codes involving neural firing and neural silence within neuron pairs are shown to efficiently encode sound information, particularly in the auditory cortex. Overall, these findings reveal a progressive transformation of the neural code from an individual, redundant, and temporal code at the periphery to a more distributed rate‐based code in the auditory cortex.

## Graphical abstract

Decoding sound from neural activity in mice reveals a striking shift in how the brain encodes sound: from precise but redundant timing codes in early auditory areas to efficient, synergistic rate‐based codes in the cortex. This transformation highlights a robust neural strategy for turning complex acoustic input into coherent perception across the auditory pathway.

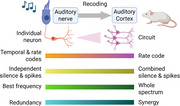

## 1 Introduction

Sound perception in natural acoustic environments can be altered by many factors, including the position and movement of both the emitter and the receiver, the presence of objects interfering with sound propagation, and the presence of competing sound sources. The neural response also depends on internal factors reflecting the instantaneous brain state, such as attention and response‐readiness.^[^ ^1^, ^2^ ^]^ For instance, cholinergic or noradrenergic neuromodulation can profoundly change the excitability of neurons and the degree of correlation between them.^[^ ^3^, ^4^, ^5^ ^]^ Despite these potentially confounding elements, the auditory system is incredibly adept at reliably identifying acoustic objects or percepts even if their presentation varies.

**Figure 1:**
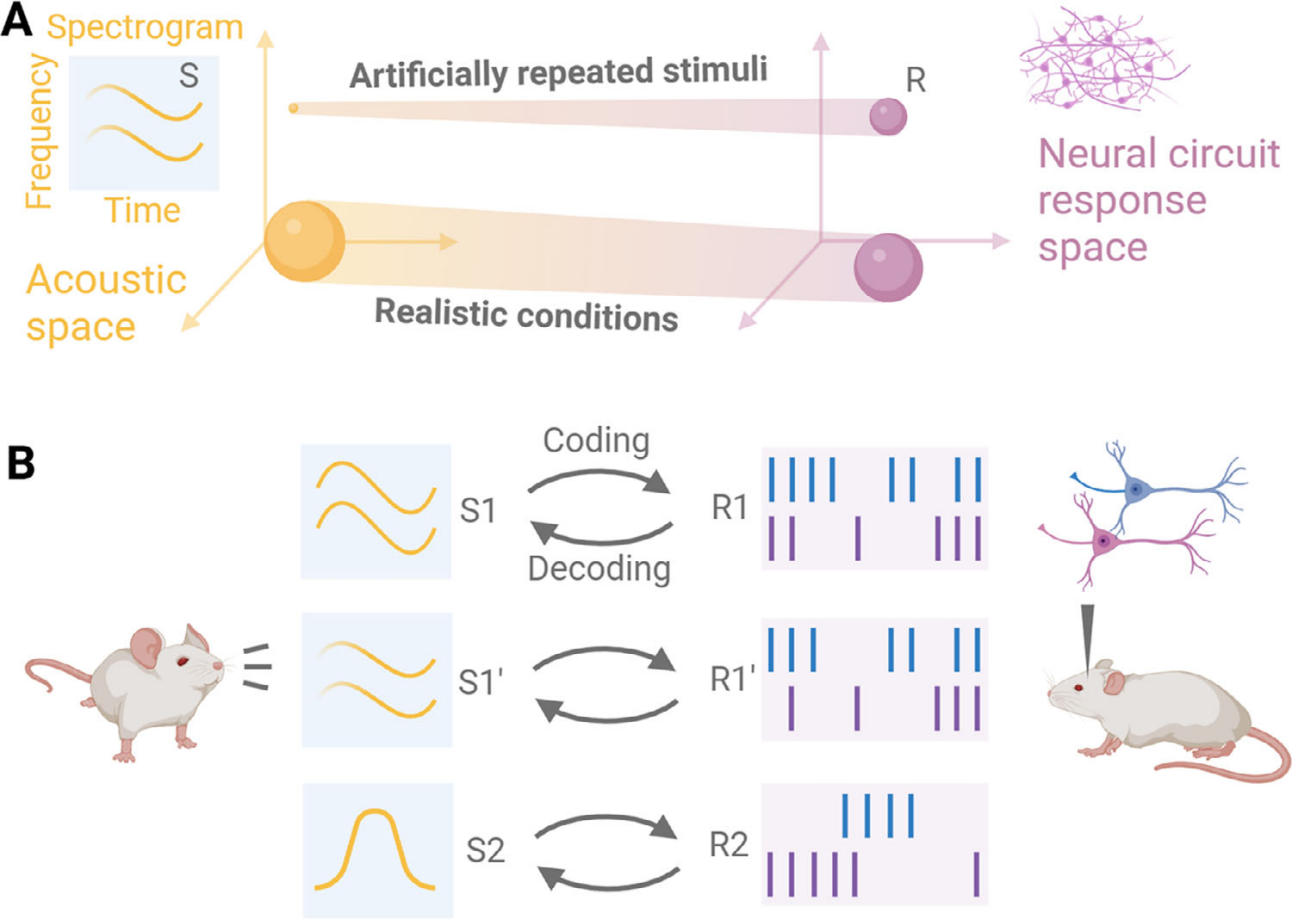
The coding/decoding scheme associating an external sensory stimulus, such as a natural mouse vocalization, with a neural response of auditory circuits. A) In classical studies, the same stimulus S is repeatedly presented and elicits a consistent, albeit slightly variable neural response, R. This is formally equivalent to finding a correspondence between a point in the sound stimulus space representing variations in acoustic features (e.g., time, frequency, energy) and a hypersphere in the space of possible responses of the neural circuits. In realistic conditions, stimuli vary due to the environment and the conditions in which the sound is emitted, but they should nevertheless elicit consistent neural responses. B) As a practical application of A), very similar stimuli (S1 and S1’) should be coded by similar evoked responses, R1 and R1’, respectively, in terms of temporal firing patterns or the number of spikes. Conversely, the observation of two similar neural circuit responses would be expected to be due to stimulation by two similar stimuli. On the contrary, stimulus S2 corresponds to a neural response R2 that is very different from R1 and R1’.

What are the implications of this for the neural code? One key property of neural codes is that they must discriminate between different inputs efficiently.^[^ ^6^ ^]^ The examples cited above suggest that another crucial property is what we refer to here as *reliability* — the maintenance of a consistent response in cases of noisy or distorted variations of a given input.^[^ ^7^, ^8^, ^9^ ^]^ In other words, with a *reliable* neural code, *similar* stimuli should elicit *similar* codes (**Figure** **1**). However, this assumption has not been demonstrated to be true and the extent to which neural codes are resistant to acoustic distortions in realistic and challenging conditions of sound emission remains unknown.

In particular, the *temporal firing patterns* (sequences of precisely timed spikes) and *firing rates* (number of spikes) of cortical neurons have been extensively studied and are widely considered to be the main components of the neural code across many sensory systems — especially in the auditory system.^[^ ^10^, ^11^, ^12^ ^]^ The information conveyed by these temporal and rate codes is typically assessed based on their discriminative power between stimuli, which critically depends on the trial‐to‐trial consistency of neural responses.^[^ ^13^, ^14^, ^15^ ^]^ This consistency — or variability — arises from intrinsic synaptic noise ^[^ ^16^ ^]^ and fluctuations in brain state ^[^ ^17^ ^]^ and therefore takes into account only the variation of the neural space (Figure 1). However, it remains unclear how robust these temporal and rate codes are to variations in the acoustic stimulus space — such as changes in source location, presence of echoes or shadows, or emitter properties (Figure 1A). In other words, it is not yet known whether these codes remain reliable when exogenous factors alter the stimulus.

Furthermore, it is also unknown whether the reliability of these codes changes along the central auditory pathways, which would provide evidence for progressive transformations in the neural code. A longstanding hypothesis suggests a transition from a temporal to a rate code as auditory information ascends the pathway.^[^ ^12^, ^18^, ^19^, ^20^ ^]^ This hypothesis is supported by studies showing a gradual degradation in both fine temporal encoding^[^ ^21^ ^]^ and trial‐to‐trial reliability^[^ ^22^, ^23^, ^24^ ^]^ along the auditory hierarchy, likely due to the biophysical properties of neurons and the temporal integration of converging inputs from one processing stage to the next. However, this view is challenged by findings of precise temporal encoding in many neurons of the auditory cortex,^[^ ^25^, ^26^, ^27^, ^28^ ^]^ which is crucial for coincident spiking — a mechanism thought to underlie ensemble‐based information encoding in the cortex.^[^ ^29^, ^30^ ^]^ These seemingly contradictory results may stem from a discrepancy between the reliability of neural codes at the single‐neuron level versus the population level. While theoretical work has highlighted the efficiency of population coding in the cortex,^[^ ^31^, ^32^ ^]^ most empirical studies have focused solely on the auditory cortex,^[^ ^33^, ^34^, ^35^, ^36^, ^37^, ^38^, ^39^ ^]^ with few attempts to compare population coding across different stages of the auditory pathway^[^ ^40^ ^]^ or to systematically contrast single‐neuron and population‐level encoding.

Here, we addressed these questions by estimating the reliability of two codes — the “temporal” code based on *temporal firing patterns* and the *“rate”* code based on firing rate — for decoding and reconstructing a random complex sound stream containing no repetitions of the same piece of sound. An individual piece of the sound stream can, therefore, be reconstructed only from neural responses to variations of this piece. We compared the reliability of the two codes along the length of the auditory pathways by taking large‐scale recordings in the inferior colliculus, medial geniculate body, and primary auditory cortex of awake head‐fixed mice and by generating responses from the auditory nerve with an advanced biophysical model. In addition to individual neuron coding, we also studied and compared population coding in these areas, as our algorithm naturally extends to populations of neurons with a sublinear time complexity.

## 2 Results

**Figure 2:**
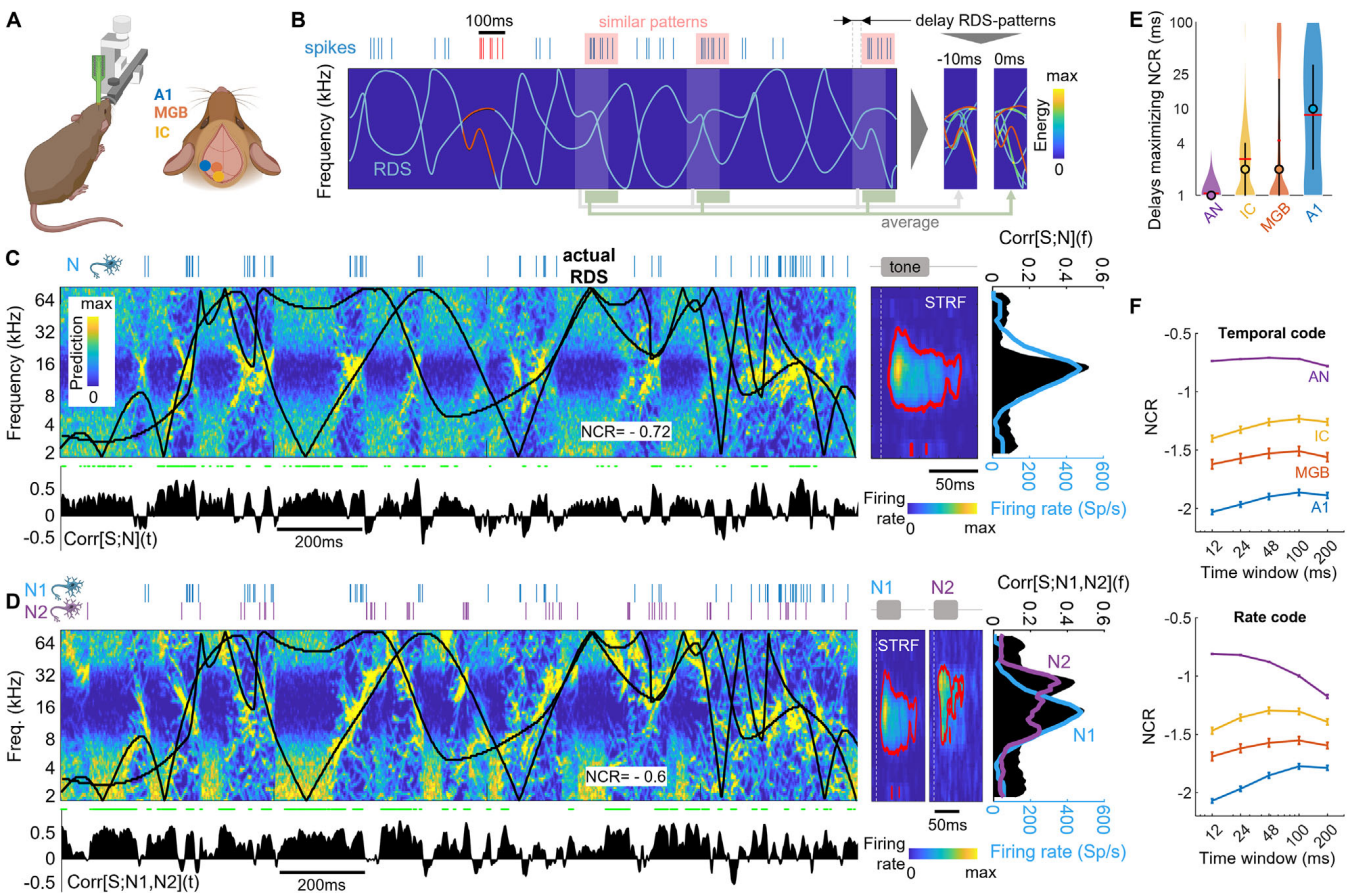
Decoding complex sound streams from spike trains. A) Schematic diagram of the recording setup for awake head‐fixed animals with implanted microelectrodes (left). Recording and craniotomy sites [inferior colliculus (IC), auditory thalamus (medial geniculate body, MGB) and primary auditory cortex (A1)] (right). B) Principle of the decoding algorithm providing a measurement of neural code reliability (NCR), here for the temporal code: (left) spectrogram (energy in time and frequency) of a Random Double Sweep (RDS).^[^ ^41^ ^]^ Recorded spikes are shown in blue above the RDS. A selected 100 ms time window of spikes is shown in red with the corresponding stimulus segment in red, preceding spikes by 10 ms to account for the biological delay between a stimulus and the neural response. Other temporal firing patterns deemed similar enough to red spikes based on Euclidean distance on convoluted spike trains are shaded in red with the corresponding RDS segments shaded in gray. (right) The corresponding stimulus segments overlaid with delays of −10 ms and 0 ms relative to the spike train are averaged, giving a prediction of the stimulus (decoding) that can be compared to the actual stimulus shown in red. C) Decoding from single‐unit recordings in the IC. (left) The black line shows the original stimulus S and the recorded spike train (the neuron N) is shown above in blue. The spectrogram shows, for each time t, the average of stimulus segments corresponding to the 100 temporal firing patterns most similar to that at time t, with colors ranging from blue (0) to yellow (max). The log_10_ of the absolute value of the correlation Corr[S;N] between the actual and decoded stimuli is our measurement of neural code reliability (NCR). Below, the correlation Corr[S;N](t) in the frequency dimension between the actual and decoded stimuli is shown in black as a function of time. (center) Spectrotemporal receptive field (STRF) of the corresponding single unit recorded. (right) Decoder performance: |Corr[S;N](f)| in the time dimension between the actual and decoded stimuli as a function of frequency in black; the maximum firing rate of the neuron extracted from its STRF is shown in violet. D) As for C, but illustrating decoding from a pair of neurons (blue and mauve). The blue neuron is identical in C and D. E) Distribution of delays maximizing NCR for single‐unit recordings compared between areas. F) On 10% of the total dataset, NCR was computed with analysis time windows of various sizes and the mean value (± standard error) is shown here for the temporal (top) and rate (bottom) codes.

We compared the strategies by which auditory neurons encode complex sounds between stages in the auditory pathway, using a large dataset of extracellular recordings (2465 multiunits gathering the activity of 3094 well‐separated single units) from 10 awake head‐fixed mice (**Figure** **2**A) in response to a complex sound stream composed of two random sweeps, the so‐called random double sweep stimulus (RDS).^[^ ^41^ ^]^ In parallel, responses to 50 ms‐long pure tones were recorded to obtain the classical auditory spectrotemporal receptive fields (STRFs). Three stages of the auditory system were probed: the midbrain (inferior colliculus (IC) [principally the central nucleus], *n* = 657 multiunits, including 835 single units), the auditory thalamus (medial geniculate body (MGB) [dorsal and ventral divisions], *n* = 907 multiunits, including 829 single units), and the primary auditory cortex ([principally A1, possibly a few units from the anterior field], *n* = 901 multiunits, including 1430 single units). In addition, 1709 single units from the auditory nerve in response to the same stimuli were obtained with an auditory nerve model adapted from that of Meddis,^[^ ^42^, ^43^ ^]^ to capture sound information coding from the cochlea to the auditory cortex.

### 2.1 A Neural Coding Reliability Algorithm for Estimating the Decoding Abilities of Individual Neurons and Groups of Neurons

We developed an algorithm for assessing the neural coding reliability (NCR) of neurons based on the principle that, with a *reliable* neural code, *similar* stimuli should elicit *similar* codes. Under this assumption, the stimulus presented to elicit a given neural response should be well predicted by averaging the 100 stimuli that triggered the most similar responses (Figure 2B). Note that the reverse process — predicting a firing rate based on similar stimulus configurations — has already been successfully applied to the vestibular system.^[^ ^44^ ^]^ This algorithm is a reverse‐correlation method^[^ ^45^, ^46^ ^]^ applied not to a single spike, but to spiking patterns with a similar temporal firing pattern, extending the notion of STRFs and making it essentially non‐linear: what is predicted for a spiking pattern is *not* a linear combination of the predictions for a single spike. Our algorithm depends on only two parameters: the time window including the spiking pattern (here 100 ms) and the distance between spike trains chosen to define “similar” spiking patterns. This makes it possible to calculate and compare different neural codes by simply changing the distance between spike trains in the algorithm. Below, we compare a *temporal code* ^[^ ^47^ ^]^ based on the Euclidean distance on convoluted spike trains (bin 0.5 ms, Hann window of length 9 ms), and a *rate code* based on the spike count within the 100 ms time window. Other distances specifically sensitive to spike timing are available^[^ ^48^, ^49^ ^]^ but are typically much more computing time‐intensive.

We estimated the accuracy of the decoded or predicted stimulus by the *NCR* metric that is NCR = *Log_10_* (*|Corr|[S;N])*, where |*Corr|[S;N]* is the absolute value of the Pearson's correlation between the predicted stimulus for the neuron *N* and the original stimulus *S* presented (Figure 2C, bottom), i.e., the correlation between these two images or, equivalently, the correlation between their vector transformed forms. While other metric choices may be possible, the NCR metric is convenient for several reasons: NCR does not depend on the FR of neurons (Figure S2Cii, Supporting Information) and can be therefore easily applied to any auditory area studied; the correlation is a computationally‐effective measure that quantifies the match between estimated and actual stimuli, a fundamental requirement of decoding methods;^[^ ^50^ ^]^ the absolute value accounts for the equal information in negative or positive correlations; the *Log* transformation reduces the strong skewness of the distribution of *|Corr|* that typically impacts the computation of usual statistics such as the mean; NCR is dimensionally equivalent to the mutual information when *S* and *N* follow a bivariate normal distribution (see supplementary material S1, Supporting Information). NCR has an approximate low‐skewed log‐normal distribution (Figure S2Ci,Ciii, Supporting Information). Throughout the manuscript, we also use the quantity *Corr[S;N](t)* (resp. *Corr[S;N](f)*) that denotes the correlation between the actual and decoded stimuli in the time (respectively frequency) dimension. This measure provides access to prediction performance at any time point *t* (respectively any frequency *f*), as shown in Figure 2C, bottom (respectively right).

The algorithm can also be used to estimate the decoding capacity of a *group of neurons* by considering the distance between the concatenated spike trains of these neurons. In the examples shown in Figure 2C,D, the prediction obtained from two neurons *N1* and *N2* clearly resembles the actual stimulus more closely than that obtained with only one of the two neurons and, consistently, *NCR* = Log_10_(*|Corr[S;N1,N2]|)* is higher.

Using NCR computations from Poisson random spike trains, we were able to establish a significance threshold for both temporal and rate codes at −2.12 (Figure S2C, Supporting Information). The time lag from the stimulus to the spike train maximizing NCR — giving the optimal decoding of complex sounds — was consistent with the classical biological time lags of auditory pathways: increasing from 1–2 ms in AN to 10–25 ms in A1 (Figure 2E). Interestingly, for high NCR values, while the time window size maximizing NCR was generally similar for temporal and rate codes in AN, IC, and MGB, it was qualitatively different in A1 with a much longer time window for the rate code (200 ms) than for the temporal code (24–48 ms, Figure S2F, Supporting Information). This result suggests that, at least in A1, the rate code requires a temporal integration of spikes over a long time window for a good decoding performance. In subsequent analyses, we chose to use a 100 ms time window for all areas to allow comparisons of NCR between auditory areas as, for this value, NCR mostly peaked or reached a plateau in the areas and for the codes studied (Figure 2F; Figure S2D, Supporting Information). We also present data for both multiunits and single units, to demonstrate that our results are not a consequence of the quality of the spike‐sorting algorithm and to provide an estimate of the decoding performance of multiunits, a spatially “local” neuron population. For the purposes of clarity, letters are used to indicate the results of statistical tests, as detailed in Table S1 (Supporting Information). Group comparisons are performed using one‐way or two‐way ANOVA tests, followed by post hoc *t*‐tests (see Experimental Section). Other statistical tests include correlation significance tests, as shown in the figures (see legends).

### 2.2 Neural Decoding Properties Change Progressively Along Auditory Pathways

**Figure 3:**
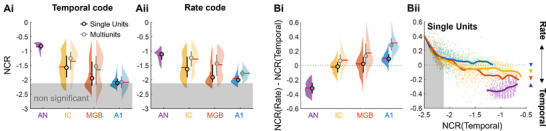
Neural code reliability over different auditory areas. For all figures, auditory nerve model data are shown in violet, recorded IC data are shown in yellow, recorded MGB data are shown in orange and recorded A1 data are shown in blue. A) NCR values for single units (black circles and lines) and multi‐units (gray circles and lines) for each auditory area for (Ai) temporal and (Aii) rate codes. B) The difference between the temporal code (Ai) and the rate code (Aii) for individual neurons is plotted in Bi with the same color codes. This difference is plotted against the NCR value for the temporal code in Bii. Bii) Solid lines are non‐linear regression lines. Significant positive correlations (*p* < 0.05, corr > 0) are shown by up‐pointing triangles and significant inverse correlations (*p* < 0.05, corr < 0) are shown by down‐pointing triangles.

We found that *NCR* decreased hierarchically along the auditory pathway, for both temporal and rate codes (**Figure** **3**Ai,Aii, tests^a,b,c,d,e^, see also Figure 2F). This decline was apparent for both multi‐ and single units. A comparison of NCR values between codes (Figure 3Bi) revealed that the decoding performance was better for the rate code than for the temporal code in A1^f^. No significant decoding performance was achieved for only 18% of neurons in A1 for the rate code, but 49% of neurons for the temporal code. More generally, the maximum information carried by an individual neuron (and a multiunit) and, therefore, the very nature of neural coding, shifted progressively along the auditory pathway, from a temporal code in the AN to a rate code in A1, the same trend being observed in multiunits and single units. This transition is consistent with the intrinsic coding capacity of neural codes. Indeed, from the IC to A1, the higher the NCR value, the greater the efficiency of the temporal code relative to the rate code (Figure 3Bii; Figure S3Ai,Aii, Supporting Information). Conversely, neurons carrying low levels of information are more accurate for the rate code. In A1, NCR values were low and the rate code was, therefore, always more efficient, on average, for stimulus decoding, than the temporal code^f^. This transition from a timing to a rate code was observed at all evoked firing rates (Figure S4Fi–Fiv, Supporting Information) and could not, therefore, be accounted for by the decrease in firing rate evoked by the RDS along the auditory pathway^g,h^ (Figure S2A, Supporting Information).

We were able to reconstruct the stereotaxic coordinates of the single units recorded. We therefore also determined whether there was a laminar gradient in NCR values. The NCR values in layer VI of A1 were significantly higher than those in layers III and V^i,j^ (Figure S3Biii, Supporting Information) and those in the ventral and medial parts of the MGB were significantly higher than that in the dorsal part^k^ (Figure S3Bii, Supporting Information).

### 2.3 Larger Receptive Fields Increase Neural Code Reliability

**Figure 4:**
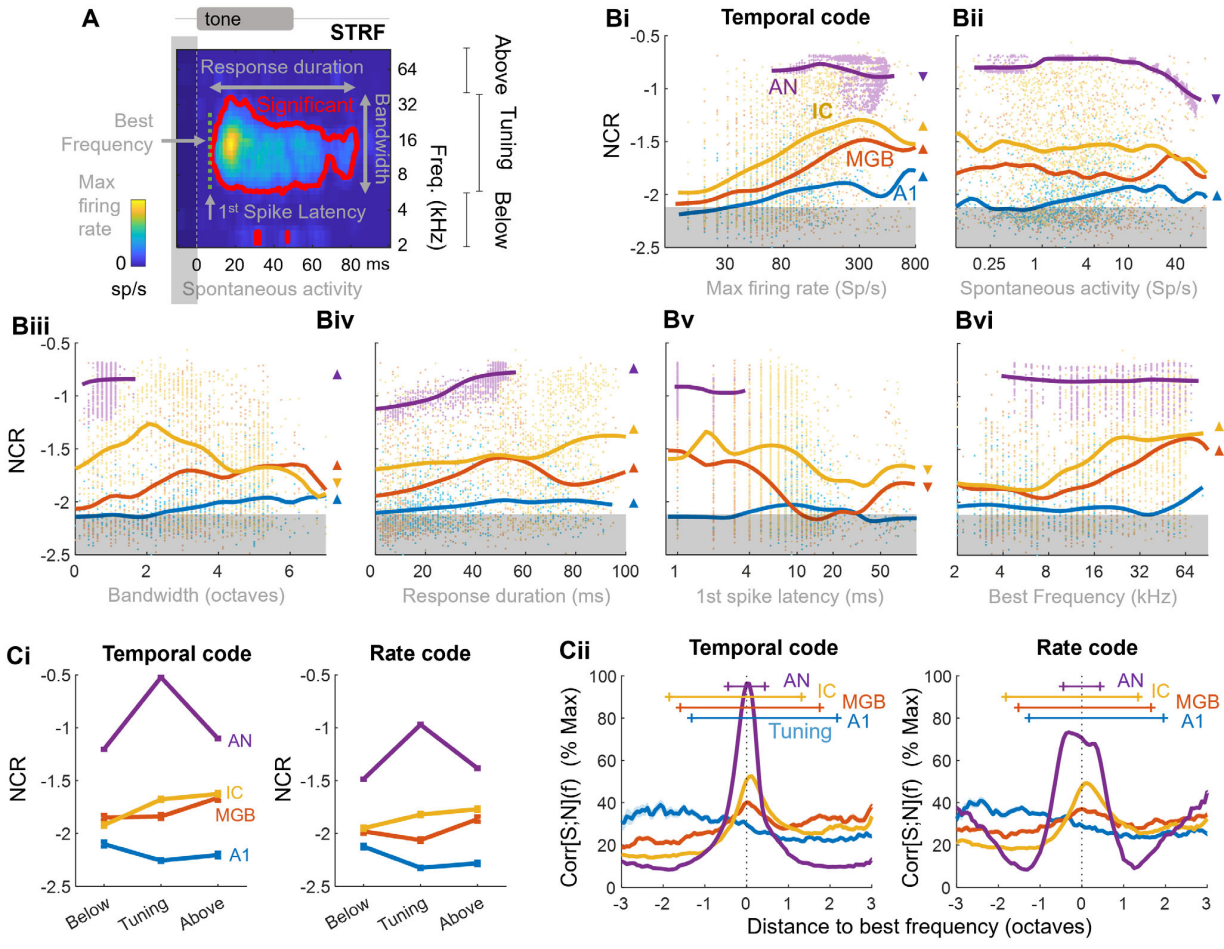
Neural code reliability and spectrotemporal receptive field (STRF) properties. A) Parameters (in gray) extracted from STRFs. B) Scatterplots of NCR values for the temporal code versus STRF parameters extracted in A). Solid lines are non‐linear regression lines. Significant positive correlations are shown by up‐pointing triangles and significant inverse correlations are shown by down‐pointing triangles. C) Relationship between the tuning of a single unit —, i.e., the frequency range of the significant response area in its STRF (A, in red) — and NCR values for both temporal (Ci, left) and rate codes (Ci, right). “Below”, “tuning” and “above” indicate the mean NCR values for frequencies below, within and above the significant area of the STRF, respectively (see A). Cii) |Corr|[S;N](f) as a function of the distance to the best frequency (BF) of the neuron for temporal (middle) and rate (right) codes. For each neuron, |Corr|[S;N](f) was divided by the maximum reached across f then centered around the best frequency of the neuron. The mean width of significant STRF peaks (i.e., tuning areas, see A) is shown at the top for each area. Ci,Cii) Only neurons with significant NCR values were considered.

We extracted several neural characteristics from the spectrotemporal receptive field (STRF) of a given neuron obtained by pure‐tone stimulation, including the spontaneous or maximum firing rate, the best frequency, frequency bandwidth, first spike latency and response duration (**Figure** **4**A; Figure S4AB, Supporting Information). For the temporal code, spontaneous activity was not related to the NCR measured in the IC and MGB, but NCR increased with the evoked firing rate in the AN, IC, MGB and A1, up to around 200–300 sp s^−1^ of the instantaneous maximum firing rate (correlation significance tests in Figure 4Bi) or 20–30 sp s^−1^ of the mean firing rate (Figure S4Di, Supporting Information), subsequently decreasing at higher firing rates. These results suggest that the NCR for the temporal code is greater for more excitable neurons, even if very high firing rates probably involve non‐evoked spike discharges and decrease the reliability of the neural response. NCR increased significantly with neural bandwidth in the AN, MGB and A1 but not in the IC, where it peaked at around two octaves (Figure 4Biii). NCR also increased with peak duration in all areas (Figure 4Biv). These results suggest that, in general, larger receptive fields (in time and frequency dimensions) are correlated with more replicable temporal firing patterns. Interestingly, neurons from the IC and MGB with shorter latencies reached higher NCR values for the temporal code (Figure 4Bv), but this effect was not significant in the AN and A1. Finally, we obtained higher NCR values for high‐frequency neurons (>16 kHz) in the IC and MGB, but not in the AN and A1 (Figure 4Bvi). Remarkably similar results were obtained for the rate code (Figure S4Ei–Evi, Supporting Information).

### 2.4 The Relationship Between Stimulus Information and Neuron Tuning Decreases Progressively Along the Auditory Pathway

In the experiments reported above, we implicitly considered the neural response to lie within significant STRF boundaries, i.e., in its tuning frequency range. However, sensory neurons can be excited by complex stimuli lying outside their STRF boundaries.^[^ ^51^, ^52^, ^53^ ^]^ We therefore investigated the reliability of neural decoding outside these boundaries. NCR values were typically greater within the STRF in the AN for both temporal and rate codes, but not in the IC^l,m,n,o^ (Figure 4Ci). In the MGB and A1, the opposite pattern was even observed, with NCR values being lower in the “tuning” frequency range than “below” or “above” it, for both types of code. This result was intriguing because, in several individual examples, the frequency profiles of NCR values closely matched the pattern of neuron firing (see the example in Figure 2C). In particular, NCR peaked at the best frequency of the neuron — the frequency at which the neuron can generate the most spikes. This phenomenon was particularly marked in the AN, consistent with results shown in Figure 4Bi, and progressively disappeared up to A1, for which the spectral profile of NCR did not peak at the best frequency but was instead biased toward lower frequencies (Figure 4Cii). Notably, in the AN, the frequency profile of NCR was heavily concentrated around the best frequency of the neuron for the temporal code, whereas the highest NCR values extended up to the flanks of the STRF for the rate code. These results suggest that the best frequency of neurons generates the most reliable spiking patterns only in the early stages of the auditory pathways, and that, on average, the reliability of the rate code gradually becomes more independent of the best frequency of neurons in the thalamocortical auditory system.

### 2.5 Fast‐Spiking Cells are Better Decoders than Regular Spiking Cells

**Figure 5:**
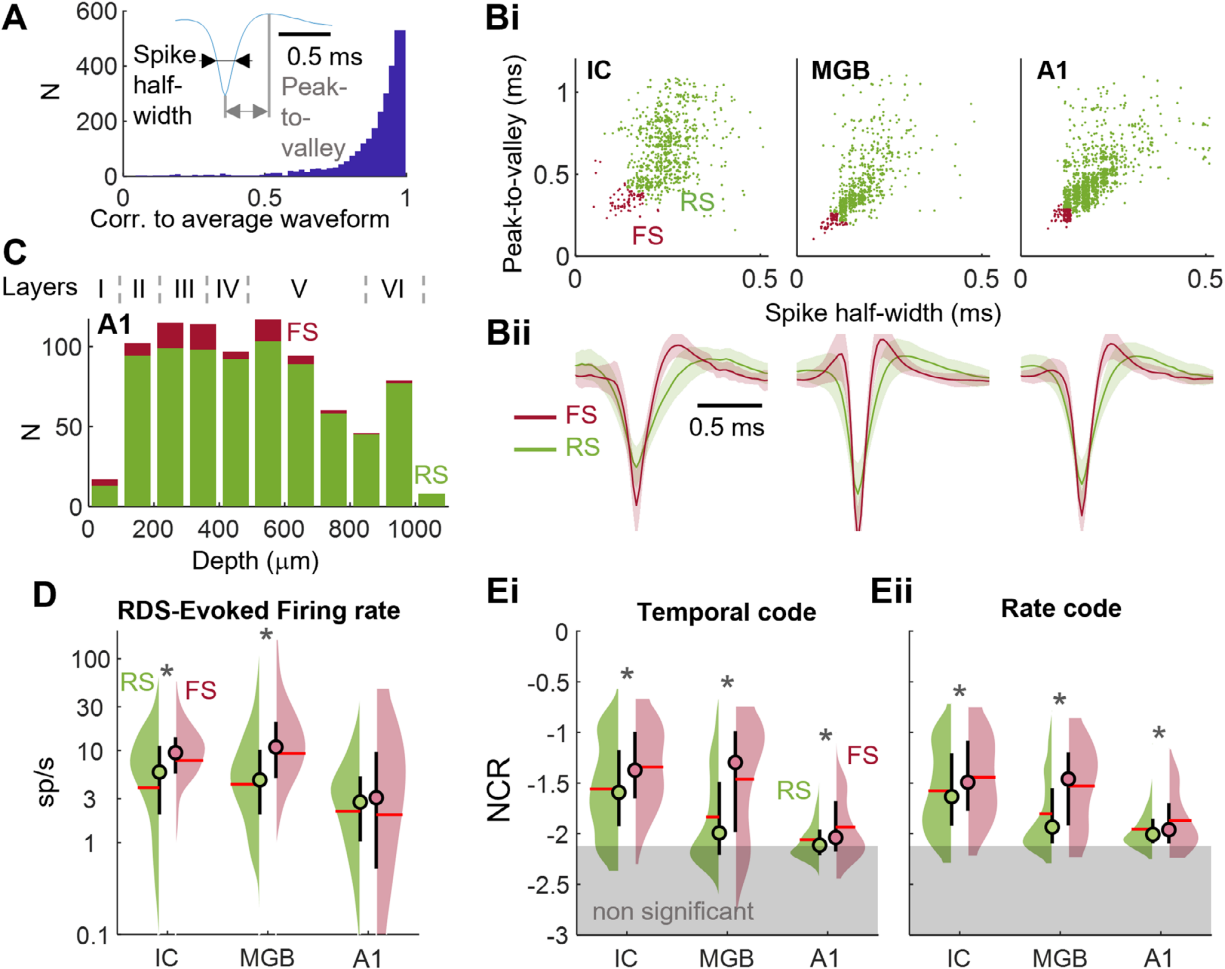
Neural code reliability in relation to neuron subtypes. A) Distribution of the correlation between waveforms and the average waveform obtained from all neurons recorded. The parameters extracted from each waveform — the spike half‐width and the peak‐to‐valley — are described in the inset. B) Scatterplot of spike half‐width versus peak‐to‐valley for A1, MGB, and IC areas (top). The 8% fastest spiking cells (FS) are colored in red whereas the remaining regular spiking cells are shown in green. Bii: Corresponding average waveforms for each group of spiking cells in A1, MGB, and IC areas. C) Laminar distribution of FS and RS cells in A1. D) Mean firing rate evoked by the presentation of the RDS stimulus: comparison between FS and RS cells. E) NCR values for FS and RS cells for both temporal (middle) and rate codes (right). D,E) stars: t‐test *p*<0.05.

Extracellular recordings span excitatory and inhibitory neurons, with inhibitory neurons accounting for about 20–30% of neurons in the lemniscal parts of the IC,^[^ ^54^ ^]^ MGB,^[^ ^55^ ^]^ and A1,^[^ ^56^ ^]^ in which our recordings were taken. About 40% of these inhibitory neurons are parvalbumin‐positive (PV^+^) in A1 and IC.^[^ ^57^, ^58^ ^]^ In A1, most PV^+^ cells are fast‐spiking (FS) cells, with a short spike half‐width and peak‐to‐valley. The other neurons in this area are typically considered regular spiking (RS) cells. After selecting spike waveforms for which these two parameters could be accurately estimated (criterion: correlation>0.4 with waveform averaged across all neurons, **Figure** **5**A), we observed a bimodal distribution of these parameters in A1 recordings (Figure 5B), according to which, we labeled as FS the cells of A1 with the shortest 8% of values for ‘2* spike half‐width + peak‐to‐valley’, a percentage consistent with that of PV cells and previous reports of FS cells in A1.^[^ ^59^, ^60^ ^]^ FS cells were more frequently found in layers II and III and the upper parts of layer V (Figure 5C). The staining profile of FS cells in the IC based on the PV marker was less clear‐cut in previous studies^[^ ^61^ ^]^ and the presence of these cells has even been called into question in the MGB^[^ ^62^ ^]^ despite the strong PV labeling in ventral areas of the MGB.^[^ ^63^ ^]^ Nevertheless, FS cells may determine functionally different cell subtypes, as in the IC,^[^ ^64^, ^65^ ^]^ so we compared NCR values for FS and RS cells in the three areas studied. To this end, we applied the same percentage criterion (8%) as in A1 for the tagging of FS cells in the IC and MGB. We found that FS cells had higher firing rates than RS cells in the IC and MGB^p,q^ (Figure 5D). We then observed that, in all three areas (IC, MGB, and A1), FS cells had higher NCR values than RS cells for both coding strategies^r^ (Figure 5Ei,Eii). In particular, there was a larger order of magnitude difference in NCR between FS and RS cells in the MGB than in other areas, suggesting that FS cells may, indeed, represent an interesting subtype of neurons in the MGB in terms of the high reliability of their response to complex sounds.

### 2.6 Firing Rate Promotes Synergy Between Neurons in the Auditory Cortex

**Figure 6:**
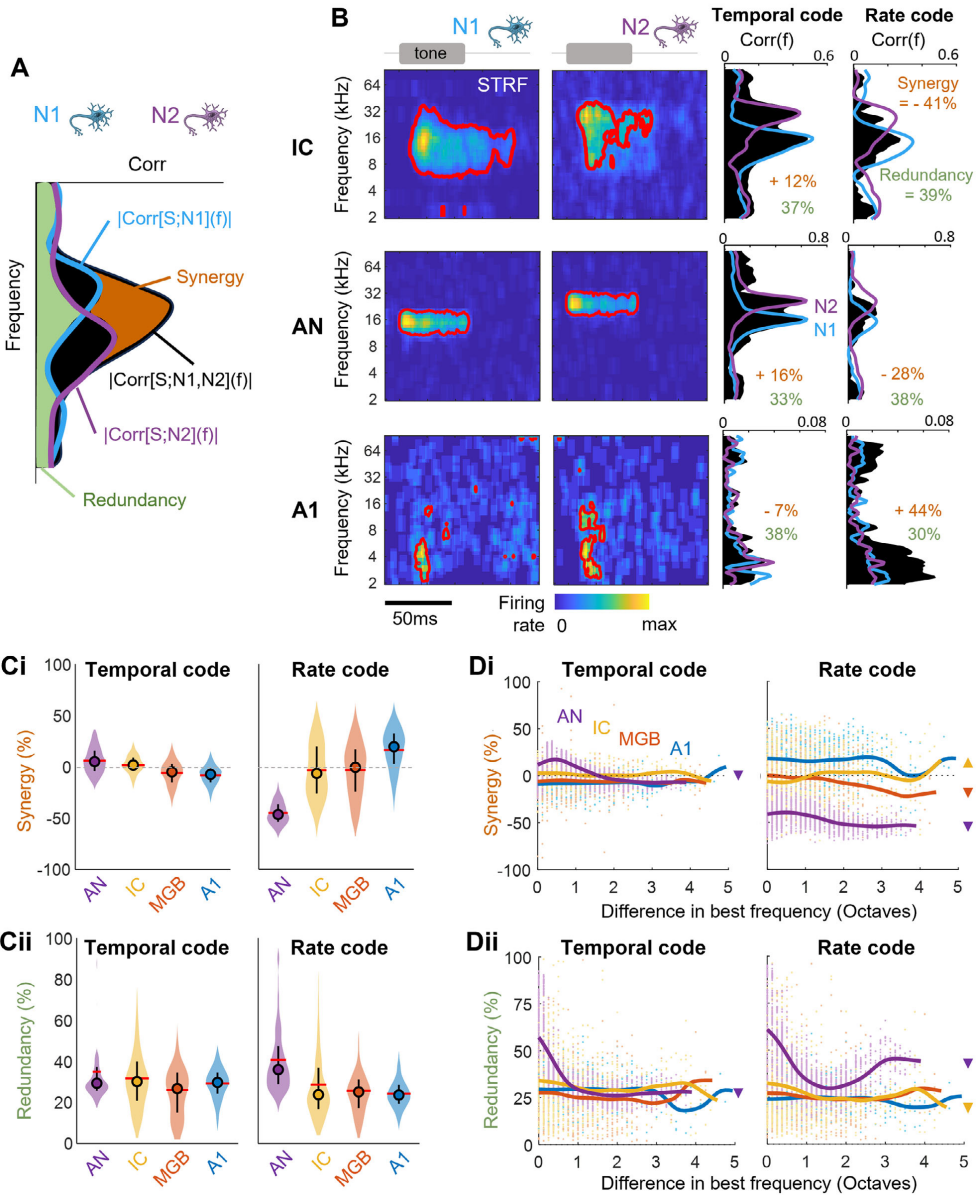
Synergy and redundancy in neural code reliability. A) Breakdown of synergy and redundancy of the NCR measured for a pair of neurons. B) Individual examples of STRFs and plots of synergy/redundancy for pairs of neurons recorded in the IC (top, same pair shown in Figure 2D), AN (middle) and A1 (bottom). C) Group results for synergy (Ci) and redundancy (Cii) for pairs of neurons along the auditory pathways and for temporal (left) and rate (right) codes. D) Synergy (Di) and redundancy (Dii) for pairs of neurons as a function of the difference in best frequency between the two neurons for temporal (left) and rate (right) codes. Solid lines are non‐linear regression lines. Significant positive correlations are shown by up‐pointing triangles and significant inverse correlations are shown by down‐pointing triangles.

NCR decreases along the auditory pathways in individual neurons, regardless of the code considered. There are, therefore, at least two ways for the brain to maintain the global amount of information: i) increasing the number of neurons, provided that the information carried by this additional population of neurons is not fully *redundant* with what is already encoded; ii) the use of a more distributed code rather than relying on individual neurons, such that the information carried by groups of neurons maintains or even adds information *synergistically* relative to the information provided by individual neurons. We investigated the strategy used by the brain, by calculating $NCR_{1,2}$ for pairs of neurons, 1 and 2, to determine the information carried by the joint responses of these two neurons (Figure 2D). As recently reported for the partial breakdown of information^[^ ^66^, ^67^, ^68^ ^]^ for a pair of neurons, information about the stimulus *S* can be provided *uniquely* by each neuron (*N_1_* and *N_2_*) separately, *redundantly* by both neurons together, or *synergistically* by both neurons together according to the following equation:

(1) $$\begin{array}{ccc} & & I\left[S;N_{1},N_{2}\right]=I_{\mathrm{Synergy}}\left[S;N_{1},N_{2}\right]+I_{\mathrm{Unique}}\left[S;N_{1}\right]\\ & & +I_{\mathrm{Unique}}\left[S;N_{2}\right]+I_{\mathrm{Redundancy}}\left[S;N_{1},N_{2}\right]=I_{\mathrm{Synergy}}\left[S;N_{1},N_{2}\right]\\ & & +I\left[S;N_{1}\right]+I\left[S;N_{2}\right]-I_{\mathrm{Redundancy}}\left[S;N_{1},N_{2}\right] \end{array}$$

where *I* is the mutual information and where *I*[*S*; *N*_1_]  = *I*_Unique_ [*S*; *N*_1_]  + *I*_Redundancy_[*S*; *N*_1_,*N*_2_]. Redundancy in this context is defined as the minimum amount of information about *S* that can be obtained from either of the two neurons.^[^ ^67^, ^68^ ^]^ Conversely, synergy is the information provided only by the two neurons together, excluding the redundant information and the information provided by each of the neurons separately. The extension of such a scheme to more than two neurons quickly runs into combinatorial problems.^[^ ^68^ ^]^ Nevertheless, this partial breakdown of information into such a structure is increasingly popular because “it implies that synergistic and redundant interactions are no longer mutually exclusive”,^[^ ^68^ ^]^ solving conceptual tensions. It is tempting to apply the same line of reasoning to predictions of *S*, measured here as the correlation |Corr|[S;N_1_,N_2_](f) between *S* and the reconstructed stimulus, and taken as a function of sound frequency *f* as in the plot on the right of Figure 2D. This correlation can then be heuristically decomposed as follows:

(2) $$\left|\mathrm{Corr}\right|\left[S;N_{1},N_{2}\right]\left(f\right)=\mathrm{Syne}\mathrm{rgy}\left[S;N_{1},N_{2}\right]\left(f\right)+\left|\mathrm{Corr}\right|\left[S;N_{1}\right]\left(f\right)+\left|\mathrm{Corr}\right|\left[S;N_{2}\right]\left(f\right)-\mathrm{Redu}\mathrm{ndan}\mathrm{cy}\left[S;N_{1},N_{2}\right]\left(f\right)$$

the equation being illustrated in **Figure** **6**A. Below, we consider a normalized version of synergy, divided by *Max*(|*Corr*|[*S*; *N*_1_,*N*_2_](*f*),|*Corr*|[*S*; *N*_1_](*f*),|*Corr*|[*S*; *N*_2_](*f*)) and then averaged over *f*. This normalized synergy reaches −100% when |*Corr*|[*S*; *N*_1_](*f*) > 0 and |*Corr*|[*S*; *N*_2_](*f*) > 0 but |*Corr*|[*S*; *N*_1_,*N*_2_] (*f*) =  0 (information is fully lost when considering the neuron pair); is equal to 0 if |*Corr*|[*S*; *N*_1_,*N*_2_] (*f*) =  *Max*(|*Corr*|[*S*; *N*_1_](*f*),|*Corr*|[*S*; *N*_2_](*f*)); and increases to 100% if |*Corr*|[*S*; *N*_1_] (*f*) = |*Corr*| [*S*; *N*_2_] (*f*) =  0 and |*Corr*|[*S*; *N*_1_,*N*_2_](*f*) > 0 (information exists only when the neuron pair is considered). An individual example is provided in Figure 6B (top), using the two same neurons as in Figure 2D. When considering information carried by the temporal code, there is little synergy (+12%) in the neuron pair as |*Corr*|[*S*; *N*_1_,*N*_2_](*f*)  ≈  *Max*(|*Corr*|[*S*; *N*_1_],|*Corr*|[*S*; *N*_2_](*f*)) . Synergy is even negative for the rate code (−41%) as |*Corr*|[*S*; *N*_1_,*N*_2_](*f*) is much smaller than |*Corr*|[*S*; *N*_1_] and |*Corr*|[*S*; *N*_2_](*f*). Conversely, redundancy is high, as the joint information closely matches the overlapping information from the two individual neurons. Similar synergy/redundancy values were obtained for the AN neuron pair shown in Figure 6B (middle) whereas the example from A1 reveals a high degree of synergy for the rate code (+44%) and no synergy for the temporal code (−7%) (Figure 6B, bottom).

We found that synergy between neurons increased considerably from the AN to the IC/MGB and then from the IC/MGB to A1 for the rate code^s,t,u^, whereas it decreased slightly along the auditory pathway for the temporal code^v,w^ (Figure 6Ci). Interestingly, synergy was greater for the temporal code in the AN and IC, whereas it was greater for the rate code in the MGB, and especially A1. There was no clear trend in terms of the variation of redundancy across areas for the temporal code^x,y^, whereas the redundancy of the rate code decreased from the AN to MGB and A1, with no significant difference between these last two areas^z,aa,ab^ (Figure 6Cii). For instance, neurons in the AN generated (weakly) synergistic spiking patterns but their firing rates were highly redundant and poorly synergistic. This result suggests that the independence of AN fibers — inherent to simulation but also biologically authentic because they are connected to different hair cells — generates very similar and reproducible firing rates but more variable temporal firing patterns between neurons.

It has been suggested that overlapping STRFs could lead to high levels of synchrony between neurons, contributing to a similarity of decoding information between neurons.^[^ ^29^, ^69^, ^70^ ^]^ We explored how the overlap of auditory receptive fields could explain the levels of synergy and redundancy over different areas observed in our data. Consistent with previous studies, we found that redundancy between neurons decreased with increasing distance between the best frequency of the two neurons in the AN, whatever the code (Figure 6Dii). Counterintuitively, this was also the case for synergy (Figure 6Di), suggesting that some level of STRF overlap was useful for providing information additional to that carried exclusively by the individual neurons of the pair. Remarkably, synergy and redundancy were less frequently dependent on the distance between best frequencies in other areas. Consistent with the results shown in Figure 4D, we provide additional evidence here that the decoding performance of individual neurons and groups of neurons progressively becomes independent of neuron tuning along the auditory pathway.

### 2.7 Neuronal Assemblies Help to Decode Sound Complexity

**Figure 7:**
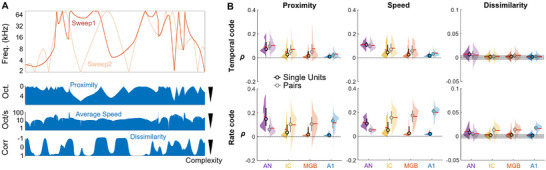
Relationship between neural code reliability and stimulus “complexity”. A) Considering two random sweeps of the stimulus (top), we derive their log‐distance (“proximity”), their mean speed, and their local correlation (“dissimilarity”), all of which are associated with a putatively greater complexity of the stimulus to be processed by the auditory pathway. B) Pearson correlation (across the time dimension) between |Corr| measurement (Figure 2C) and proximity (left), average speed of the stimuli (middle) or dissimilarity (right), for temporal (top) and rate (bottom) codes. The gray area indicates a lack of significant correlation (see Figure S7, Supporting Information).

We then investigated the possible functional role of this synergy between pairs of neurons in the neural code. There is a consensus that complex features of sound stimuli that are ambiguous at the individual neuron level may be better dealt with by increasing the dimensionality of the neural representation —, i.e., by using neural assemblies.^[^ ^71^, ^72^ ^]^ We tested this hypothesis by extracting three features describing the complexity of our stimulus as a function of time. First, if two random sweeps are very close (“Proximity” feature, see **Figure** **7**A and Experimental Section), it is potentially difficult for a single neuron to resolve their simultaneous fine variations because both sweeps excite the same synaptic inputs. Second, it is assumed that stimulus complexity increases with the mean speed of the two sweeps (“Average speed” feature). Third, stimulus complexity increases for highly negative local correlations indicating strong opposite variations over time (“Dissimilarity” feature), as illustrated in Figure 7A. We observed that the correlation between NCR and our three complexity measures generally i) increased along the auditory pathway for pairs of neurons for the rate code; ii) decreased along the auditory pathway for single units for the rate code and for single units and pairs for the temporal code^ac,ad,ae,af,ag,ah,ai^ (Figure 7B–D). NCR was significantly greater for neuron pairs than for individual neurons in the IC, MGB, and A1, but smaller in the AN (except for proximity with the temporal code), whatever the code^aj^. These results suggest that part of the complexity of the stimulus may be progressively decoded by the simultaneous firing of neuron assemblies rather than by the activity of individual neurons.

### 2.8 Neural Silence Significantly Contributes to Neural Coding in Subcortical Pathways

**Figure 8:**
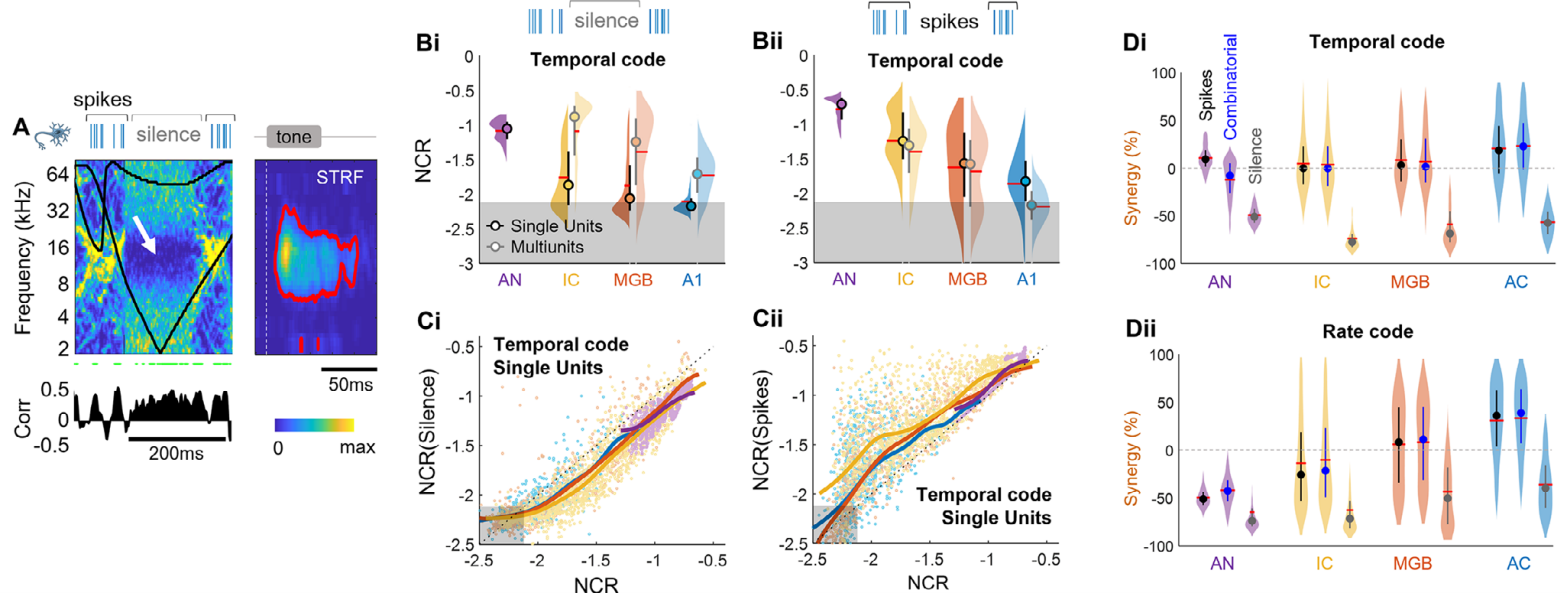
Decoding reliability of neural silence and spikes. A) Extract from Figure 2C focusing on a time interval including neural silence. The blue band (white arrow) indicates that most stimulation segments associated with a silent neuron never included tones in the frequency range corresponding to the significant response of the neuron in its STRF shown on the right. As a consequence, the decoded stimulus predicts that the actual RDS (in black) had sweeps outside this blue band, which was indeed the case. Bi) NCR for single units (black circles and lines) and multiunits (gray circles and lines) as a function of area for the temporal code when considering only periods of neural silence. Bii) As in Bi but considering only time intervals with spikes. C) NCR for neural silence (Ci) and when considering only time intervals with spikes (Cii) plotted against NCR for the whole spike train. B,C) Solid lines are non‐linear regression lines. The gray area corresponds to non‐significant NCR values. D) Synergy for pairs of neurons considering only spikes, neural silence or a combination of spikes on one channel and silence on the other (“Comb.”), for the temporal (Di) or rate (Dii) codes. Synergy is always calculated relative to the NCR measured for a pair of neurons including the whole spike train, as in Figure 6A.

It is typically assumed that neural coding is carried by spikes, but our findings highlight the carriage of a significant amount of information about the stimulus in *neural silence*, that is, in the *absence* of spikes. When looking at stimulus prediction (Figure 2C), we were intrigued by the presence of blue bands during periods of neural silence (**Figure** **8**A, white arrow). These blue bands indicate that it is *unlikely* that one of the two random sweeps crossed the STRF of the neuron during neural silence (see legend of Figure 8A). This true prediction leads to the measured NCR being highly positive during periods of silence.

Does this phenomenon occur in all areas? We found that the decoding ability of neural silence decreased along the auditory pathway^ak,al,am^, becoming non‐significant for most neurons (65%) in A1 (Figure 8Bi). In addition, the NCR calculated for periods of neural silence was strongly related to, and systematically lower than the NCR computed on the whole spike train (Figure 8Ci), suggesting that auditory neurons are generally as reliable for generating spikes as for remaining silent. The silence‐related NCR values obtained for multiunits were markedly higher than those for single units^an^, as it is less likely and, therefore, more reliable to observe simultaneous neural silence for all the component units of the multiunit. This can be illustrated by the individual example in Figure 2D for the pair of neurons: the correlation between prediction and the actual stimulus (and therefore the NCR) is larger for the neuron pair than for the single neuron in comparisons of the corresponding periods of neural silence between Figure 2C,D.

But does neural silence disturb neural coding? We measured NCR restricted to periods of spiking and excluding periods of neural silence (Figure 8Bii). We found that the information exceeded that calculated from the whole spike train^ao^, especially for NCR< ‐1, which corresponds to low information values (Figure 8Cii). Single units and multiunits provided a similar range of NCR values in the IC and MGB (but not in A1), suggesting that the difference in NCR between single units and multiunits shown in Figure 3Ai results mostly from the inclusion of neural silence in the IC and MGB spike trains. Nevertheless, the silence‐related NCR for multiunits was significantly greater^ap^ than that obtained from spikes alone in the IC, MGB and A1 (Figure 8Bi,Ci), suggesting that the observations of silence simultaneously on several different channels may be a reliable decoder, possibly even as reliable as simultaneous firing.

We assessed the decoding efficiency of local neural assemblies including neural silence in more detail by investigating whether synergy emerged from silence on a pair of single units, or from a combinatorial code^[^ ^73^, ^74^, ^75^, ^76^ ^]^ with one neuron firing while the other remains silent, and we compared this synergy to that achieved when both neurons fire simultaneously (Figure 8D). First, the silence of both neurons was not synergistic (and could even have a negative effect) for both codes and all areas, whereas a combinatorial code was as synergistic as both neurons firing simultaneously, for both codes and in all areas^aq,ar,as,at^ (except for the temporal code in the AN). Second, and consistent with the results shown in Figure 6Di, the synergy between the firing rates of neurons increased considerably from a negative average in the AN and IC to a positive average in the MGB and A1 for the combinatorial code or the firing of two neurons simultaneously^aq,ar,au,av^.

Overall, our results provide evidence that neural silence carries significant information in subcortical pathways, and that the silence of one neuron while another neuron is firing can serve as a synergistic neural code in A1.

## 3 Discussion

**Figure 9:**
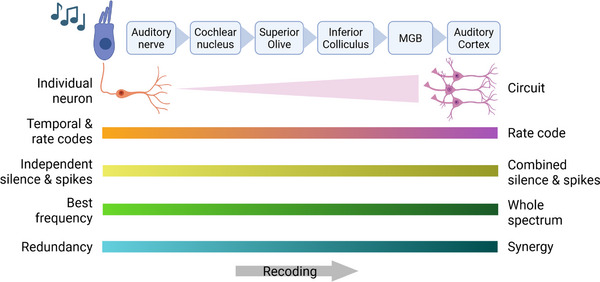
Putative progressive recoding along auditory pathway.

The following global picture emerges from our results (**Figure** **9**): firing rate and temporal firing patterns contain reliable information about the stimulus in the AN, but only the rate code remains significantly reliable when progressing from the subcortical auditory areas to A1. For both codes, the information carried peaks at the preferred tuning frequency of the neuron in the AN but become progressively independent in the receptive fields of later stages of the auditory pathway. This recoding is accompanied by a transfer of the reliability of neural responses from individual neurons to groups of neurons. In A1, specifically, these groups of cells are synergistic for the rate code, carrying more information about fine acoustic features together than as individual neurons. Along the auditory pathway, neural silence displayed significant reliability for the decoding of complex sounds, especially in individual neurons in the AN and neural assemblies in A1.

### 3.1 Time Windows for Information

Our algorithm extends the concept of receptive fields by providing i) non‐linear stimulus‐response functions; and ii) dynamic predictions, which vary over time. This resolves one of the main criticisms against linear receptive fields: their inability to account for the non‐linear response properties of auditory neurons,^[^ ^77^, ^78^, ^79^ ^]^ during responses to complex sounds, for example, when autocorrelation prevails. One of the limitations of our method is the need to choose a time window for analysis. For comparisons between areas, we chose a 100 ms window for all neurons because this duration gave the overall highest values of NCR across all areas. However, this limitation also made it possible to compare optimal time windows for information between areas. Interestingly, for the rate code, the optimal time window increased from 12 ms in the AN to 100 ms in the MGB and A1. This is consistent with the (empirical) idea that short timescales facilitate representations of rapidly fluctuating stimuli in the periphery whereas longer timescales make it possible to combine information in temporally separate inputs and to form consistent noise‐robust representations of sensory objects in the cortex.^[^ ^80^ ^]^ The value of 100 ms in A1 is consistent with the 20–100 ms range found for maximum discrimination abilities in the MGB and A1^[^ ^81^ ^]^ and with the 64 ms window used in a similar study,^[^ ^40^ ^]^ but is a bit longer than the 10–50 ms range found in A1 in ferrets.^[^ ^15^ ^]^ However, it is likely that the neurons within a cerebral area have a range of different optimal time windows, endowing them with different functional properties, as has been suggested for the human auditory cortex.^[^ ^82^ ^]^ Taking this idea further, according to the theory of multiplexed codes, multiple neural codes operating at different timescales would encode complementary stimulus features.^[^ ^11^ ^]^ This possibility merits a more thorough investigation of the distribution of optimal time windows in each area and its relationship to neuron decoding abilities.

### 3.2 Reliability of Individual Neurons Along the Auditory Pathway

The idea that firing rate carries information about stimuli is seminal to neuroscience, but the idea of a temporal code, with the precise times at which spikes occur also carrying significant information, is more recent.^[^ ^83^, ^84^, ^85^, ^86^ ^]^ Typically, temporal and rate codes may be important if the stimuli can be discriminated on the basis of the timing of spikes or the total spike count, respectively.^[^ ^25^, ^87^ ^]^ Temporal and rate codes may actually coexist in individual neurons or at the scale of a neural population.^[^ ^11^, ^16^, ^25^, ^88^, ^89^ ^]^ We first investigated the reliability of these two codes at the single neuron level.

We found that the reliability of individual neurons strongly decreased along the auditory pathway, reaching a level just above that expected by chance in A1, consistent with the increase in trial‐to‐trial variability along the auditory pathway.^[^ ^22^, ^23^, ^24^ ^]^ Several factors can explain the decrease in NCR for both codes. First, variations of brain state^[^ ^23^, ^90^ ^]^ are assumed to have a more dramatic impact on the trial‐to‐trial variability of cortical networks than subcortical networks. Second, the spectrotemporal integration of sounds involves increasingly nonlinear processing along the auditory pathway,^[^ ^91^, ^92^, ^93^ ^]^ making it harder, or even impossible to associate pieces of the sound spectrogram to sequences of spikes. For instance, if we take the extreme case of a single spike, which is used to build classical STRFs by reverse correlation methods, there is a significant difference between the STRF estimated from pure tones and that due to natural stimuli in A1, preventing any reliable decoding from a single spike.^[^ ^94^, ^95^, ^96^, ^97^ ^]^ As a second example, the simultaneous increase in neural adaptation along the auditory pathway, facilitating the formation of noise‐tolerant neural representations, crucially limits the reliability of neural responses by forward suppressing or delaying spikes for longer periods of time at later stages in the auditory pathway.^[^ ^98^, ^99^ ^]^

The rate code became progressively more reliable than the temporal code along the auditory pathway (Figure 3Aiii). This may appear surprising, because many studies have shown temporal firing patterns to be better than firing rate at discriminating natural sounds, such as vocalizations,^[^ ^25^ ^]^ probably because spike timing makes use of a higher dimensional space than firing rate to describe the neural response. We show here that temporal firing patterns may achieve intrinsically higher decoding abilities (Figure 3Bii), consistent with previous studies,^[^ ^30^, ^100^ ^]^ but that high decoders relying on the temporal code are much more frequent in individual neurons of the subcortical pathways than in A1. This view is consistent with the idea that there is a transition from a temporal to a rate code along the auditory pathway.^[^ ^12^, ^18^, ^19^ ^]^ Spike timing can remain very precise even in A1,^[^ ^27^, ^28^ ^]^ so our results suggest that a degradation of the reproducibility of the whole sequence of spikes rather than its distortion by spike jittering may be at stake here. This recoding is assumed to support noise‐tolerant representations^[^ ^99^ ^]^ (but see^[^ ^22^ ^]^), to reduce redundancy between neural activities^[^ ^40^ ^]^ and to support better discrimination between complex inputs.^[^ ^101^ ^]^ Our findings support this hypothesis by showing that the complexity of sounds is more efficiently decoded by the temporal firing patterns and firing rates of individual neurons in the AN and by the firing rate of pairs of neurons in A1 (Figure 7).

### 3.3 Reliability of Neurons in Terms of Functional Properties

The information rates of both the temporal and rate codes increased with evoked firing rate, consistent with theory.^[^ ^16^ ^]^ Other theoretical considerations based on Fisher information^[^ ^102^, ^103^ ^]^ or stimulus‐specific information^[^ ^104^, ^105^ ^]^ have suggested that the maximum information about stimuli carried by the firing rate of neurons may change between the high‐firing rate (peak) and high‐slope (flanks) regions of the auditory receptive field with decreasing noise levels or with increasing neural population size.^[^ ^14^, ^105^ ^]^ Our observations are globally consistent with this view. Indeed, NCR for the rate code peaked at the flanks (and best frequency) of STRFs in the AN, as expected, but only at best frequency in the IC and MGB (Figure 4Dii), possibly due to higher noise levels, as suggested by Butts and Goldman.^[^ ^105^ ^]^ We obtained two other major findings. First, NCR always peaked at the highest firing rate (i.e., best frequency) for the temporal code, indicating that the reliability of temporal firing patterns is greatly affected by the decrease in firing rate on the STRF flanks. Second, reliability became progressively independent of tuning along the auditory pathway, for both codes. For instance, in the IC or MGB, tuned parts of the STRF had moderate NCR values, particularly relative to the (high) frequency range above tuning (Figure 4Di). Indeed, consistent with the predictions of the above models, the best decoded stimuli were typically less clearly defined for the broader and fuzzier STRF at later stages in the auditory pathway.^[^ ^14^ ^]^ This may be because our results suggest that these stages favor a population coding for the acoustic features of sounds. For instance, a complex narrowband sound encoded by a pair of neurons with non‐overlapping STRFs would lead to one of the neurons firing outside of its receptive field.

### 3.4 Individual Versus Population Coding, Redundancy Versus Synergy

This line of reasoning supports the fundamental idea that additional functions emerge in populations of neurons for the processing of information beyond what can be achieved with single neurons.^[^ ^80^ ^]^ Consistently, we observed that pairs of neurons or multiunits gave higher levels of prediction than individual neurons (Figure 3Ai; Figure S6D, Supporting Information). Indeed, it has been almost universally shown in neuroscience that a high variance of neural firing rates is typically captured by a very small number of the neurons recorded.^[^ ^37^, ^40^, ^106^, ^107^ ^]^ Conversely, a small number of neurons may be sufficient to elicit small but measurable changes in sensory perception or motor behavior.^[^ ^108^ ^]^ With increasing numbers of neurons, the proportion of the variance explained is generally bounded by the (noise) correlation of neural activities,^[^ ^71^, ^107^, ^109^, ^110^ ^]^ emerging from similar tuning in particular, due to common inputs.^[^ ^111^, ^112^ ^]^ This was the case in our study for the MGB and A1, in which the recorded neurons lie on a laminar and, therefore, isofrequential dimension, and in which the NCR becomes saturated for groups of neurons larger than multiunits, contrasting with the IC, in which electrode penetration spans a wide range of the tonotopical axis (Figure S6D, Supporting Information).

We assessed the synergy of neuron pairs as a metric easier to interpret than signal and noise correlations. The two approaches are related: higher signal correlations reflect redundancy as each neuron provides similar information, and some configurations of noise correlation may reduce overlap and provide synergy.^[^ ^50^, ^80^ ^]^ Here, since our NCR metric is based on correlations rather than information theory, we constructed measures of synergy and redundancy by mimicking the equations used in partial information decomposition.^[^ ^66^, ^67^, ^68^ ^]^ These measures exhibit the expected behavior, thereby enabling meaningful comparisons with the existing literature. In general, the peripheral parts of sensory systems are considered to display high levels of redundancy at the populational level^[^ ^113^ ^]^ to ensure tolerance to misfiring and noise or the overrepresentation of specific salient features.^[^ ^76^ ^]^ However, this generates high metabolic needs, which may not be compatible with the functioning of neocortical columns. Redundancy decreased along the auditory pathway for firing rate, although to a much lesser extent than in the seminal study by Chechik et al.^[^ ^40^ ^]^ Both our study and that by Chechik et al. showed that redundancy between similarly tuned neurons was high in the AN, whereas it was less dependent on the best frequency difference between neurons in the MGB and A1 (Figure 6E). We showed that this was also the case for synergy. Surprisingly, synergy values were distributed around 0 for the temporal code in all areas (Figure 6Di). This suggests that, unlike firing rates, temporal firing patterns combine in a somewhat linear way between neurons in the auditory pathway, in a non‐synergistic but also non‐destructive manner, as synergy values were not negative, at odds with the theory of pattern‐based coding.^[^ ^74^ ^]^ In any case, the large variance of synergy values for both rate and temporal codes when considering only spikes or combinatorial codes (Figure 8Ci) reveals the existence of highly synergistic pairs for both rate and temporal codes. This supports the coexistence of synergistic and redundant hubs in specialized subnetworks for combining the advantages of maximizing encoded information through synergy and offering a robust backbone through redundancy.^[^ ^80^, ^114^ ^]^

### 3.5 Global Information in Auditory Areas

At population level, it remains unclear how the brain progressively recodes the stimulus along the auditory pathway while maintaining the overall level of information about the stimulus. A decrease in NCR in individual neurons between two auditory stages may require a compensatory increase in the number of neurons. There are ten times more neurons in the IC than in the AN (at least in rats^[^ ^115^, ^116^ ^]^), consistent with the order of magnitude difference in NCR between the AN and IC. However, the magnitude of the decrease in redundancy between the IC and A1 shown here and reported in the study by Chechik et al. is smaller than the increase in the size of the neuron population between these two areas. Consequently, global redundancy among neurons probably increases from the subcortical to the cortical parts of the pathway, given the differences in population size between these two areas, as already pointed out by Barlow.^[^ ^117^ ^]^ In any case, this model does not hold for the MGB. Indeed, in our study, the MGB was positioned systematically between the IC and A1 in terms of decoding performance or redundancy, whereas it has only a third the number of neurons present in the central IC and about an eighth the number of neurons present in A1.^[^ ^115^, ^118^ ^]^ More information recoding may occur in the MGB than previously thought, in ways that remain to be elucidated and continue to be a matter for debate.^[^ ^18^, ^119^, ^120^ ^]^ For instance, non‐synchronized firings,^[^ ^121^ ^]^ dynamic shaping through communication loops with A1^[^ ^119^ ^]^ and plasticity upon learning^[^ ^122^, ^123^ ^]^ may be part of a large repertoire of recoding strategies in the MGB.

Recoding strategies in A1 are also far from fully understood. A possible neural code may use higher‐order statistics of neuronal responses.^[^ ^111^, ^124^, ^125^ ^]^ For instance, heterogeneous nonlinear mixtures of selectivity represent an efficient way to increase the dimensionality of a population code, allowing an easy readout of the population code by simple linear decoding^[^ ^6^, ^126^, ^127^ ^]^ and potentially better predictive behavior.^[^ ^128^ ^]^ Another important code relies on sparse representations,^[^ ^57^, ^108^, ^129^, ^130^, ^131^ ^]^ in which small and specific groups of active neurons represent complex inputs,^[^ ^26^, ^132^, ^133^ ^]^ possibly building on the massive excess of thalamic inputs in A1 and, therefore, the overcompleteness of the code. Population codes (and sparse codes in particular) necessarily imply the firing of some neurons at a given time whilst others remain silent, raising questions about the amount of stimulus information carried by neural silence. We found that silent neurons individually carry little information and certainly much less than spikes, a result consistent with a previous study in the peripheral visual system.^[^ ^76^ ^]^ However, we observed that combining the silence of one neuron with temporal firing patterns or firing rate of the other neuron in a pair led to comparable synergy to combining the spiking activity of both neurons and much greater synergy than the combination of two silent neurons (Figure 8C). Similar observations have been reported for the peripheral and cortical visual systems, and the olfactory system.^[^ ^73^, ^74^, ^75^, ^76^ ^]^ Here, this kind of combinatorial code proved highly synergistic in A1, contrasting with earlier stages of the auditory pathway.

How do circuits recognize neural silence and use it as a code? They may be sensitive to neural silence if silent neurons are connected to inhibitory neurons feeding forward onto readout neurons.^[^ ^76^, ^134^ ^]^ For instance, sparse coding may result from an excitation‐inhibition balance biased in favor of inhibition, with a strong involvement of PV^+^ interneurons.^[^ ^135^ ^]^ However, silence and sparseness have limitations. For example, the near‐synchronous activation of tens to hundreds of cells is required to drive downstream neurons, so the involvement of too few neurons in the response to a stimulus might end the transfer of information through brain circuits.^[^ ^108^ ^]^ Further investigations of large‐scale recordings are warranted to determine whether there is a saturation or decline of information as the proportion of silent neurons increases.

## 4 Conclusion

Our results highlight the profound transformation of the neural code along the auditory pathway. They support the idea that sound information is progressively transferred from highly precise temporal firing patterns in independent neurons at the periphery, to synergistic neuronal networks that primarily use firing rate at the cortical level. Intermediate coding strategies appear in the midbrain and thalamus, reflecting a gradual shift between these two modes of representation. Our results also suggest a progressive diminution of the role of tonotopic organization in the lemniscal system as information reaches the upper stages. The algorithm designed here for analyzing coding in the auditory pathway could readily be extended to other neural systems. This approach may therefore yield new insights into the features of the codes of individual neurons and populations of neurons elsewhere in the central nervous system.

## 5 Experimental Section

### Subjects

Recordings were performed in CBA mice from Janvier Laboratories between postnatal days 61 and 184. All animals were housed in an animal facility with controlled humidity (50–55%) and temperature (22–24 °C) conditions, under a 12 h light/12 h dark cycle (lights on at 7:00 am) with free access to food and water. All aspects of this study were approved by the Institut Pasteur animal research ethics committee.

### Surgery

Animals were moved to the surgical room 24 h before acute experiments. Before surgery, anesthesia was induced with medetomidine (Domitor at 1 mg kg^−1^) followed by the inhalation of isoflurane gas (0.8% to 1% isoflurane at a flow rate of 0.7 L min^−1^ in 95% oxygen). Animals were also given buprenorphine (Vetergesic, 0.1 mg kg^−1^) by intraperitoneal injection for analgesia 30 min before craniotomy. Animals were given meloxicam (Metacam, 5 mg kg^−1^) and glucose (0.5%) subcutaneously immediately after surgery and once daily for the next three days.

Following induction, the animals were shaved and headfixed with a mask that both delivered the gaseous mixture for the maintenance anesthesia and stabilized the head for the determination of stereotactic coordinates and for craniotomy. Topical lignocaine (laocaine, 10 mg kg^−1^) was administered subcutaneously above the skull and the surgical site was cleaned with povidine iodide (Vetegesic Solution). The skin was opened by a scalpel blade incision through the midline. The skin around the craniotomy site was removed and Vetbond was applied to hold it in place. Stereotactic coordinates from the Paxinos and Franklin mouse brain atlas^[^ ^136^ ^]^ were subsequently mapped relative to Bregma with Lambda and vasculature surface markings used to ensure correct demarcation for the primary auditory cortex (A1), median geniculate body (MGB) and inferior colliculus (IC). No more than two areas were exposed in any given animal. A small additional craniotomy was performed on the contralateral side for use as a ground electrode entry point, using either a metal pin or screw. For longer term (chronic) experiments, animals were also fitted with a custom‐designed head post for headfixation, positioned anterior to bregma. A small 3D printed grid was placed over the auditory cortex to reduce swelling. The head post and skull were subsequently covered in dental acrylic cement (Super Bond, Phymep) leaving the craniotomy window open. The craniotomy window was subsequently filled with KWIK‐CAST (World Precision Instruments).

### Recordings

Chronic headfixed recordings were performed in animals treated with the anxiolytic medetomidine (Domitor 0.1 mg kg^−1^) for about 1 h. Extracellular recordings were performed with 32‐channel laminar silicon probes (Neuronexus, A1×32‐Poly2‐10mm‐50s‐177‐CM32). Warm saline (0.9% NaCl) was systematically applied to the surface of the exposed brain.

One probe was implanted at a time, vertically, at the coordinates listed in the Table 1 below:

**Table 1:** The location of implanted probes.

Location	Anteroposterior [µm]	Mediolateral [µm]	Depth [µm]
IC re lambda	850–1150	850–1150	475–1600
MGB re bregma	4750–4900	2200–2750	2200–4000
A1 re bregma	2350–2650	4500–5000	225–1500

In A1, depth coordinates were corrected for the difference between the 30° angle (to the vertical) of the micromanipulator and the 50° angle of the line perpendicular to the brain surface at the A1 location. It was also assumed that the tissue was pushed inward by 100 µm. Layer boundaries were then chosen in accordance with the proportions described in other studies.^[^ ^137^ ^]^

Data were acquired with a Plexon digitizing amplifier (DA1/32) and an acquisition board (OmniPLex D) at a sampling rate of 40 kHz. Recording sessions were performed in awake animals for 1 – 3 h at a time, but multiple sessions were performed for any given animal.

The acquired signal was analyzed with in‐house routines in Matlab (Mathworks). Extracellular recordings were high bandpass‐filtered above 600 Hz then thresholded at three standard deviations above the signal to extract action potential times and associated waveforms. Single units were sorted with KiloSort 3 applied to concatenated signals from STRF and RDS stimulation, to ensure that the same single units were studied with the two protocols.

### Sounds

All sounds were delivered with RZ6 hardware (Tucker Davis Technologies (TDT)) connected to a free‐field electrostatic ES1 speaker (TDT) placed 2 cm from the ear canal contralateral to the craniotomy. Sounds were generated at a sampling rate of 195.312 kHz. The speaker was calibrated with noise to estimate its transfer. The inverted transfer function was applied to all sounds sent to the speaker. The speaker produced a flat spectrum (± 3 dB) between 2 and 90 kHz after calibration.

Spectrotemporal receptive fields were determined with 45 gamma‐tone frequencies (the product of a gamma distribution and sinusoidal tone), covering 5.5 octaves (2–90 kHz), presented in a random order at a rate of 4.15 Hz and at 75 dB SPL. Each tone was presented eight times. If STRFs did not appear sharp enough or convincingly auditory no protocol was run. Otherwise, the random double sweep (RDS) stimulus was presented.^[^ ^41^ ^]^ Briefly this stimulus consisted of two continuous pure tone voices with a random frequency modulation at a speed <10 Hz over a period of 5 min. At the end of the protocol, another STRF was obtained to confirm the stability of the recording. The animals were returned to their cages at the end of the recording session and were killed by cervical dislocation three to six weeks after the recording session.

In each session, the frequency response area was also determined with the same set of tones as the STRF presented from 75 to 5 dB SPL (5 dB steps, random order), at a rate of 2 Hz. Each tone was presented eight times at each intensity. These frequency response areas were calculated as for STRFs (see below) and were used to check that mice had normal auditory thresholds (data unshown).

### Building STRFs

The STRFs derived from single‐ or multi‐unit activity were obtained by constructing post‐stimulus time histograms for each frequency, with 1 ms time bins. All STRFs were smoothed with a uniform 5×5 bin window. The best frequency was then defined as the frequency at which the highest firing rate was recorded. A significant peak in the STRF was defined as a firing rate contour above the mean level of baseline activity (estimated from the first 10 milliseconds of STRFs) plus six times the standard deviation of the baseline activity. For a given site, “bandwidth” was defined as the sum of all peak widths in octaves.

### RDS Processing

Custom‐developed Matlab codes were ran, reproducing the algorithm described in Figure 2 on the spike times of single units, multiunits, pairs of single units, and groups of all the single units or multiunits recorded. The code used the extended computation capacities of the HPC Core Facility of Institut Pasteur.

### Auditory Nerve Model

In addition to extracellular recordings, 1709 single units were simulated from the auditory nerve in response to the same STRF and RDS stimuli, using an auditory nerve model adapted from the work of Meddis.^[^ ^42^, ^43^ ^]^ Computational details have been described elsewhere.^[^ ^19^, ^42^ ^]^ This model was adapted to the data by matching the proportion of low‐ middle‐ and high‐firing rates of the simulated AN fibers, using published data for mice.^[^ ^138^ ^]^ The distribution of best frequencies of simulated AN fibers were also matched with the global distribution of best frequencies across all areas that were encountered in the data (Figure 5Bvi).

### Statistical Analysis

Mostly, ANOVA (one‐way, two‐way, three‐way) was used to test for effects in the data. Stimulus parameters were systematically considered to be categorical in ANOVA. If significant ANOVA results were obtained, post‐hoc Student's *t*‐tests were performed, with Tukey–Kramer correction for multiple comparisons. Tests were two‐tailed. Several parameter values (such as NCR) were recorded for a limited number of animals, but could not use a nested analysis because the animal factor was not nested in the area factor as most animals were recorded in several areas.

The statistical distribution of many parameters, including firing rates and NCR, is typically skewed. Therefore a log_10_ transformation was applied to render these distributions less skewed. The robustness of ANOVA to small deviations from normality^[^ ^139^, ^140^ ^]^ and the large sample sizes of the groups (see Table S1, Supporting Information) ensured that ANOVA was a valid option. Furthermore, there is currently no satisfactory non‐parametric solution for two‐way and three‐way tests. An alpha level of 0.05 was used for all statistical tests.

Violin plots use the Matlab toolbox developed by B. Bechtold (https://doi.org/10.5281/zenodo.4559847).

## Conflict of Interest

The authors declare no conflict of interest.

## Author Contributions

A.B. and T.D. contributed equally to this work. B.G. performed conceptualization; A.B. and T.D. performed data acquisition; A.B. and T.D. performed animal preparation; B.G., A.B., and R.A.C. performed the formal analysis; J.B., J.‐L.P., N.M., and B.G. acquired resources; B.G., A.B., and T.D. wrote the original draft. All authors reviewed and edited the final manuscript; J.‐L.P., B.G., and N.M. performed supervision, project administration, and funding acquisition. All the authors have read and agreed to the published version of the manuscript.

## Supporting information



Supporting Information

## Data Availability

All datasets and custom MATLAB code used to reproduce the figures, supplementary figures and statistical tests in the paper are freely available at https://doi.org/10.5281/zenodo.16323433, hosted on Zenodo. The NCR algorithm code is openly accessible via a public GitLab repository (https://gitlab.pasteur.fr/ida‐public/ncr‐algorithm.git) and is also archived in the Zenodo release.
