# Supplementary material for: Neural Response Reliability as a Marker of the Transition of Neural Codes along Auditory Pathways
Source: Adv Sci (Weinh). 2025 Sep 18;12(46):e08777. doi: 10.1002/advs.202508777 (PMC12697799; doi:10.1002/advs.202508777)
Supplement: Supplementary file 1 — Supporting Information [file ADVS-12-e08777-s001.docx]

Supplementary Materials

| Ref | Test | Stat value | p-value |
| --- | --- | --- | --- |
| a* | Anova 1 way, area, temporal code, Single units | F(3,4799)=2974 | <10^-10^ |
| b* | Anova 1 way, area, temporal code, Multi-units | F(2,2462)=516 | <10^-10^ |
| c* | Anova 1 way, area, rate code, Single units | F(3,4799)=1520 | <10^-10^ |
| d* | Anova 1 way, area, rate code, Multi-units | F(2,2462)=424 | <10^-10^ |
| e | T-tests area 1 vs area 2, temporal and rate code, all areas, single units and multiunits |  | <10^-10^ |
| f | Paired t-tests NCR temporal vs rate codes, A1, single units and multiunits |  | <2.5x10^-3^ |
| g | Anova 1 way, area, RDS evoked firing rate, single units | F(3,4281)=1988 | <10^-10^ |
| h | T-tests area 1 vs area 2, RDS evoked firing rate, all area pairs except MGB vs IC, single units |  | <10^-10^ |
| i* | Anova 1 way, A1 layers, temporal and rate code, Single Units | F(5,823)>4 | <1.3x10^-3^ |
| j | t-tests NCR temporal code, layers VI vs III and V, Tukey’s correction for multiple tests |  | <0.04 |
| k* | Anova 1 way, MGBv vs MGBd, temporal and rate code, Single Units | F(1,792)>12,8 | <4.10^-4^ |
| l* | Anova 2 ways, area * frequency range, temporal code, Single units, factor area * frequency range | F(6,11298)=276 | <10^-10^ |
| m | T-tests within STRF vs below or above, temporal code, single units, Tukey’s correction for multiple tests, all areas |  | <10^-10^ |
| n* | Anova 2 ways, area * frequency range, rate code, Single units, factor area * frequency range | F(6,12704)=190 | <10^-10^ |
| o | T-tests within STRF vs below or above, temporal code, single units, Tukey’s correction for multiple tests, all areas |  | <10^-10^ |
| p | T-tests RDS-evoked firing rate, RS vs FS cells, unequal variance, IC, MGB |  | <10^-4^ |
| q | T-test RDS-evoked firing rate, RS vs FS cells, unequal variance, A1 |  | 0.72 |
| r | T-tests NCR temporal and rate code, RS vs FS cells, unequal variance, all areas |  | <0.014 |
| s* | Anova 1 way, area, rate code, Single units | F(3,4474)=1924 | <10^-10^ |
| t | T-tests AN vs IC, MGB vs A1, rate code, single units, Tukey’s correction for multiple tests |  | <10^-10^ |
| u | T-test IC vs MGB, rate code, single units, Tukey’s correction for multiple tests |  | 1 |
| v* | Anova 1 way, factor area, Synergy, temporal code, Single units | F(3,4004)=290 | <10^-10^ |
| w | T-tests area 1 vs area 2, temporal code, single units, Tukey’s correction for multiple tests |  | <4 x10^-3^ |
| x* | Anova 1 way, factor area, Synergy, temporal code, Single units | F(3, 4004)=70 | <10^-10^ |
| y | T-tests, area 1 vs area 2, temporal code, single units, Tukey’s correction for multiple tests |  | <3x10^-3^ |
| z* | Anova 1 way, factor area, Synergy, rate code, Single units | F(3,4478)=356 | <10^-10^ |
| aa | T-tests AN vs IC, IC vs MGB, IC vs A1, temporal code, single units, Tukey’s correction for multiple tests |  | <10^-10^ |
| ab | T-test MGB vs A1, rate code, single units, Tukey’s correction for multiple tests |  | 0.22 |
| ac* | Anova 1 way, factor area, Proximity, Average Speed or Dissimilarity, temporal code or rate code, Single units or pairs |  | <10^-10^ |
| ad | T-test area 1 vs area 2, Proximity, temporal code, single units or pairs, Tukey’s correction for multiple tests, except MBG vs IC (pairs) |  | <0.05 |
| ae | T-test area 1 vs area 2, Proximity, rate code, single units or pairs, Tukey’s correction for multiple tests, except MBG vs IC (single units+pairs) and A1 vs MGB (pairs) |  | <0.05 |
| af | T-test area 1 vs area 2, Average Speed, temporal code, single units or pairs, Tukey’s correction for multiple tests |  | <0.05 |
| ag | T-test area 1 vs area 2, Average Speed, rate code, single units or pairs, Tukey’s correction for multiple tests |  | <0.05 |
| ah | T-test area 1 vs area 2, Dissimilarity, temporal code, single units or pairs, Tukey’s correction for multiple tests, except A1 or MBG vs IC (single units) and A1 vs IC (pairs) and MGB vs AN (pairs) |  | <0.05 |
| ai | T-test area 1 vs area 2, Dissimilarity, rate code, single units or pairs, Tukey’s correction for multiple tests, except IC vs MGB (pairs) |  | <0.05 |
| aj | T-test all areas, Dissimilary or Proximity or Average Speed, temporal or rate code, pairs > single units (A1, MGB, IC) or single units > pairs (AN) |  | <0.05 |
| ak* | Anova 1 way, area, temporal code, Single units | F(3,4798)=2333 | <10^-10^ |
| al* | Anova 1 way, area, temporal code, Multi-units | F(2,2456)=332 | <10^-10^ |
| am | T-tests area 1 vs area 2, temporal and rate code, all areas, single units and multiunits |  | <10^-7^ |
| an | t-tests NCR temporal code, silence, single units vs multiunits, unequal variance, IC, MGB and A1 |  | <10^-10^ |
| ao | Paired t-tests NCR temporal code, spikes only vs all activity, all areas, single units |  | <10^-10^ |
| ap | t-tests NCR temporal code, silence multiunits vs spikes only single units, unequal variance, IC, MGB and A1 |  | <10^-10^ |
| aq | Anova 2 ways, area * neuron pair code, temporal code, Single units, factor area * neuron pair code | F(6,12009)=222 | 0 |
| ar | T-test spikes vs combinatorial code, temporal code, Tukey’s correction for multiple tests, all areas except AN |  | p>0.05 |
| as | Anova 2 ways, area * neuron pair code, rate code, Single units, factor area * neuron pair code | F(6,13400)=168 | 0 |
| at | T-test spikes vs combinatorial code, rate code, Tukey’s correction for multiple tests, all areas except AN |  | p>0.05 |
| au | T-test spikes, rate code and combinatorial code, Tukey’s correction for multiple tests, AN vs IC; IC vs MGB; MGB vs A1 |  | p<0.05 |
| av | T-test average > 0, rate code and combinatorial code, rate code, MGB & A1 |  | p<10^-10^ |

*: we did not use a nested analysis because the animal factor was not nested in the area factor as some animals were recorded in several areas.

*Supplementary Table 1 : Statistical tests*

*Supplementary Material 1: Note on the NCR metric*

If the joint distribution of the stimulus S and the predicted stimulus N is bivariate normal, then the mutual information *I[S;N]* is related to the correlation coefficient *Corr[S;N]* by ^[1]^

$$I\left[ S,N \right]=-\frac{1}{2}\log\left( 1-{Corr}^{2}\left[ S;N \right] \right)$$

$$=\frac{1}{2}\log\left( \frac{1}{1-{Corr}^{2}\left[ S;N \right]} \right)$$

When the correlation is very small, as in our study, this expression can be approximated as:

$$I\left[ S,N \right]\approx\frac{1}{2}\log\left( {Corr}^{2}\left[ S;N \right] \right)$$

$$\approx\log\left( \left| Corr \right|\left[ S;N \right] \right)$$

$$\approx log(10)\times NCR$$

Bibliography

[1] I. M. Gelʹfand, *Calculation of the Amount of Information about a Random Function Contained in Another Such Function*, American Mathematical Society **1959**.

*Supplementary Figure 1 :*


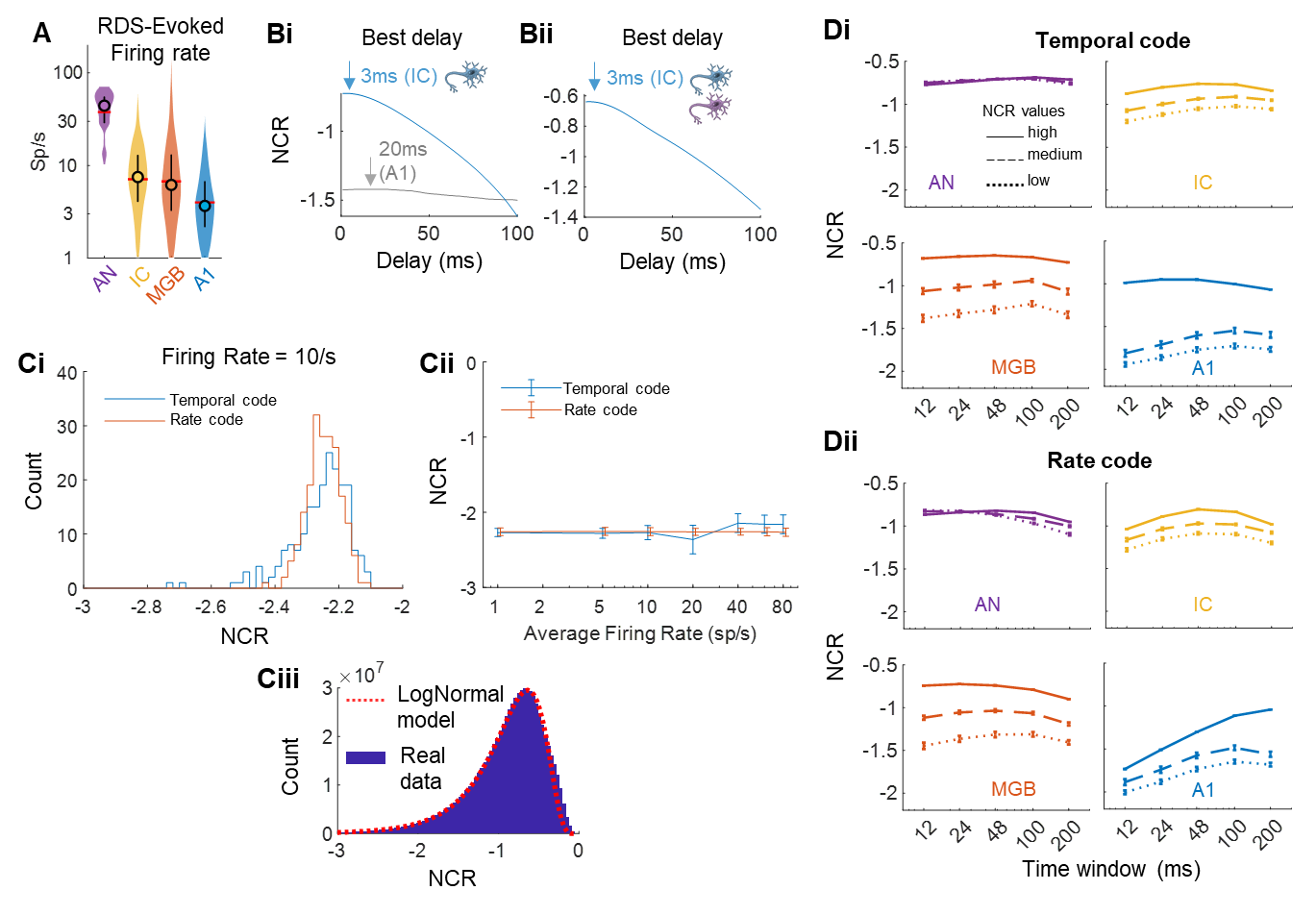


*Supplementary Figure 2 : A) Mean firing rate evoked by the presentation of the RDS stimulus. B) NCR as a function of the delay between the stimulus and the time window of neural activity considered for Bi) the example of Figure 2C in the IC and an example in A1 with a longer best delay and Bii) the example of Figure 2D. C) Computation of a significance threshold for NCR. Ci) Distribution of NCR values for both temporal and rate codes using simulated Poisson spike trains at 10 spikes per second (close to the average rate observed on RDS recordings, see A). Cii) Mean NCR for simulated Poisson spike trains as a function of the firing rate. NCR does not depend on firing rate. From Ci and Cii, the 99.5% percentile of all NCR values for all simulated values of firing rate was taken as a significance threshold for NCR, i.e. -2.12. Ciii) Distribution of NCR(t)=Log10(|Corr|[S;N](t)|) values for all neurons and all times of the RDS stimulus. Fitting by a Log Normal model is superimposed (dashed red line). D) As in Figure 2F, NCR as a function of the size of the analysis time window shown for Di) temporal and Dii) rate codes. Curves were separated in three groups of NCR values: the third lowest values of the distribution (“low”), the third higher (“high”) and the remaining values (“medium”).*


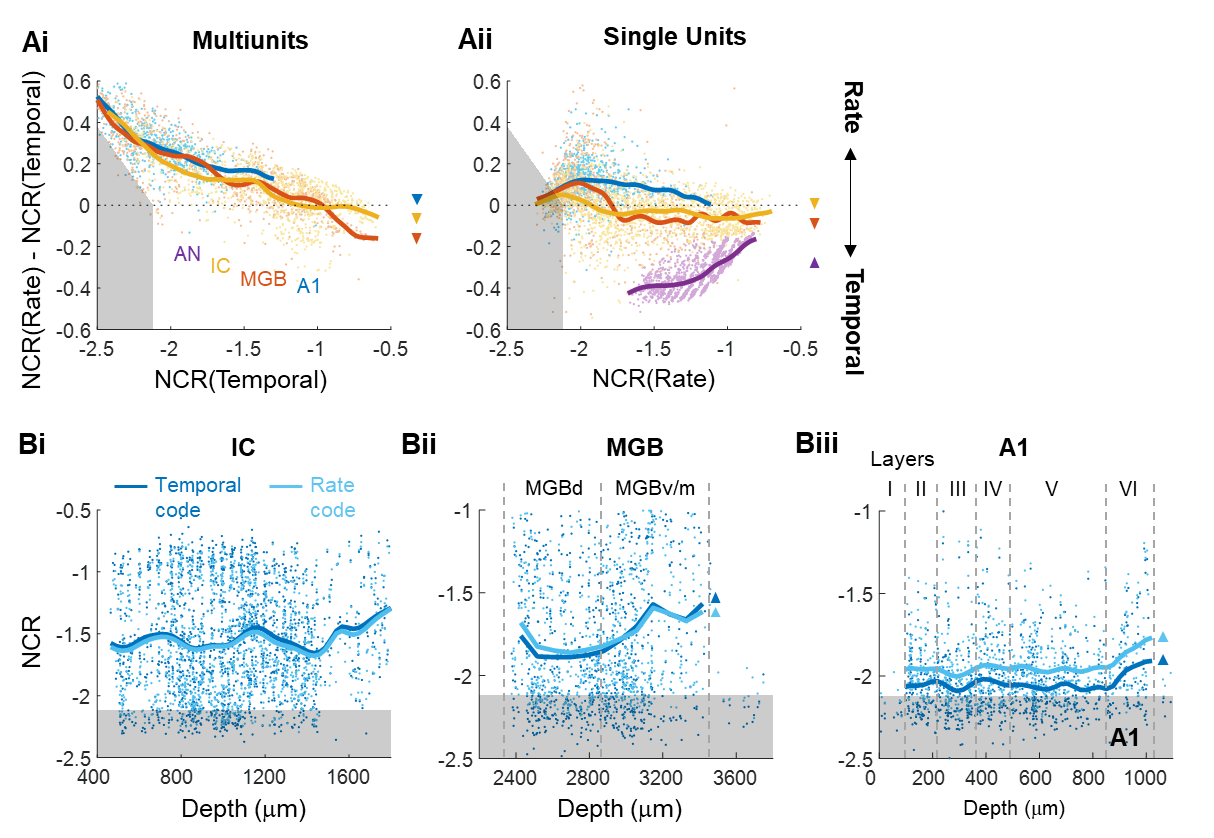


*Supplementary Figure 3 : Ai) Difference between temporal and rate codes for a given neuron is plotted against the NCR value for the temporal code. Aii) As in Ai against the NCR value for the rate code. B) Mean NCR as a function of recording depth in Bi) the IC, Bii) the MGB and Biii) the A1 for both codes. A,B) Solid lines are non-linear regression lines. Significant positive correlations are shown by up-pointing triangles and significant inverse correlations are shown by down-pointing triangles.*


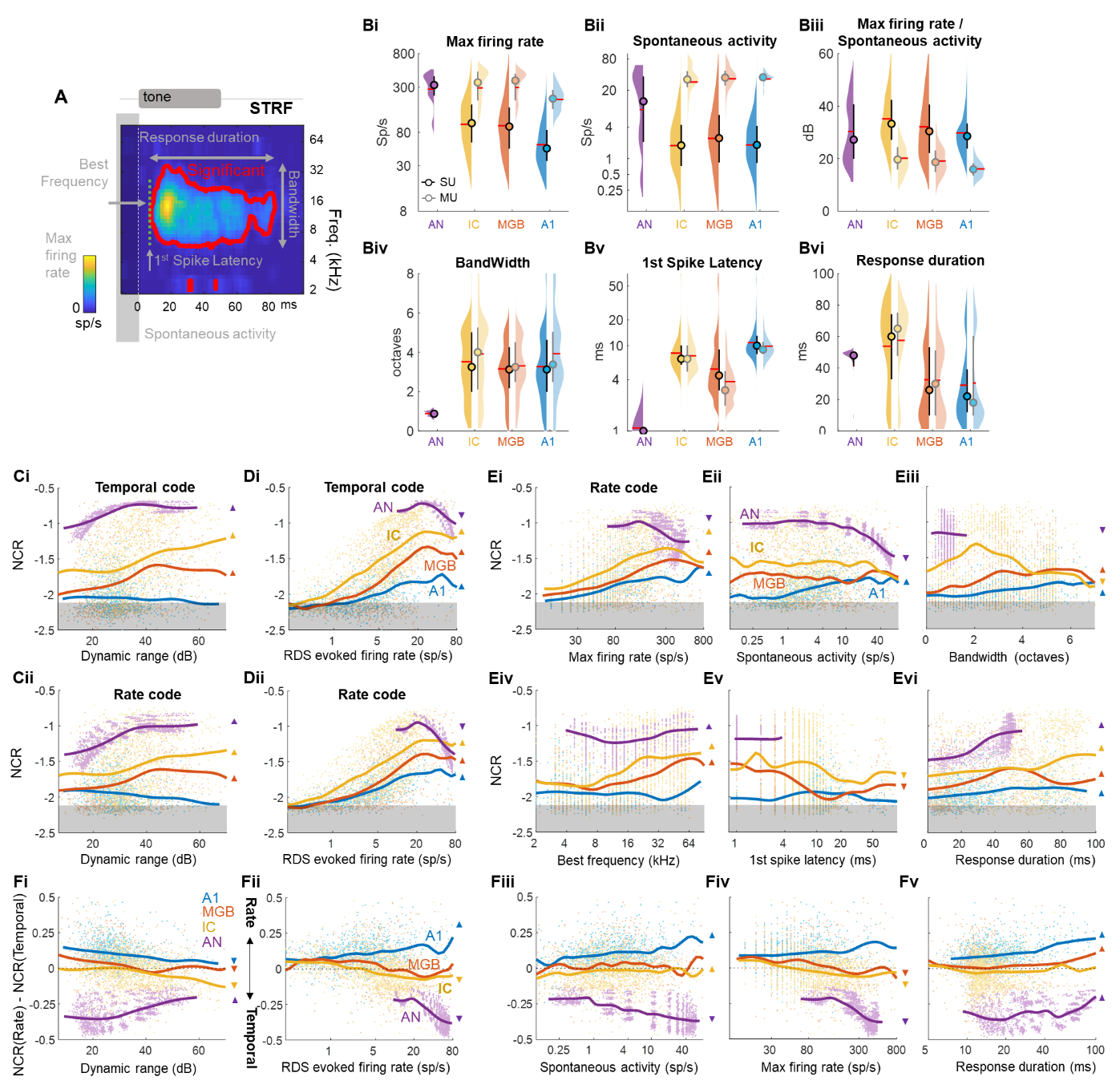


*Supplementary Figure 4 : A,B) Tuning properties of neurons. A) Example of a spectrotemporal receptive field in the IC: the average evoked firing rate is plotted as a function of frequency of the pure tone in ordinate and time after the pure tone in abscissa. Red contour is the significant peak of the STRF (see methods). Tuning properties of a given neuron are then characterized by parameters extracted from this STRF and illustrated here in green: maximum firing rate, best frequency (frequency of the maximum firing rate), spontaneous activity (mean firing rate on the time range [-10 0]ms), dynamic range as the ratio between this maximum firing rate and the spontaneous activity, peak bandwidth, first spike latency, and peak duration. B) Distribution of these parameters on the whole database of recordings for single- and multi-units. C-F) Complementary analysis to Figure 5: C) Scatterplots of NCR versus dynamic range values for Ci) temporal and Cii) rate codes. D) same as C for the RDS evoked firing rate. E) Same as Figure 5B for rate code. F) Difference of NCR values for temporal and rate codes as a function of Fi) the dynamic range, Fii) the firing rate evoked by RDS presentation, Fiii) the spontaneous activity of neurons, Fiv) the maximum firing rate of neurons, Fv) the peak duration of STRFs. Solid lines are non-linear regression lines. Significant positive correlations are shown by up-pointing triangles and significant inverse correlations are shown by down-pointing triangles.*


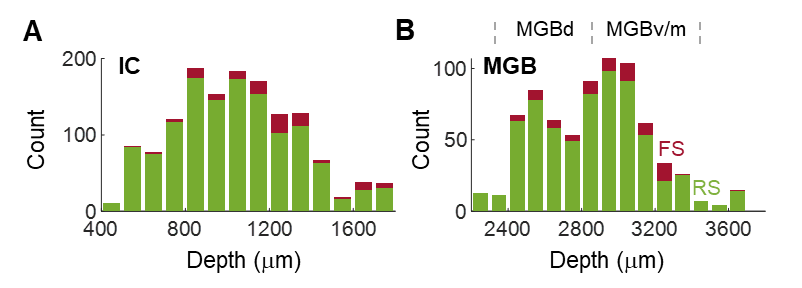


*Supplementary Figure 5 : Laminar distribution of fast spiking (FS) and regular spiking (RS) cells in A) the IC and B) the MGB.*


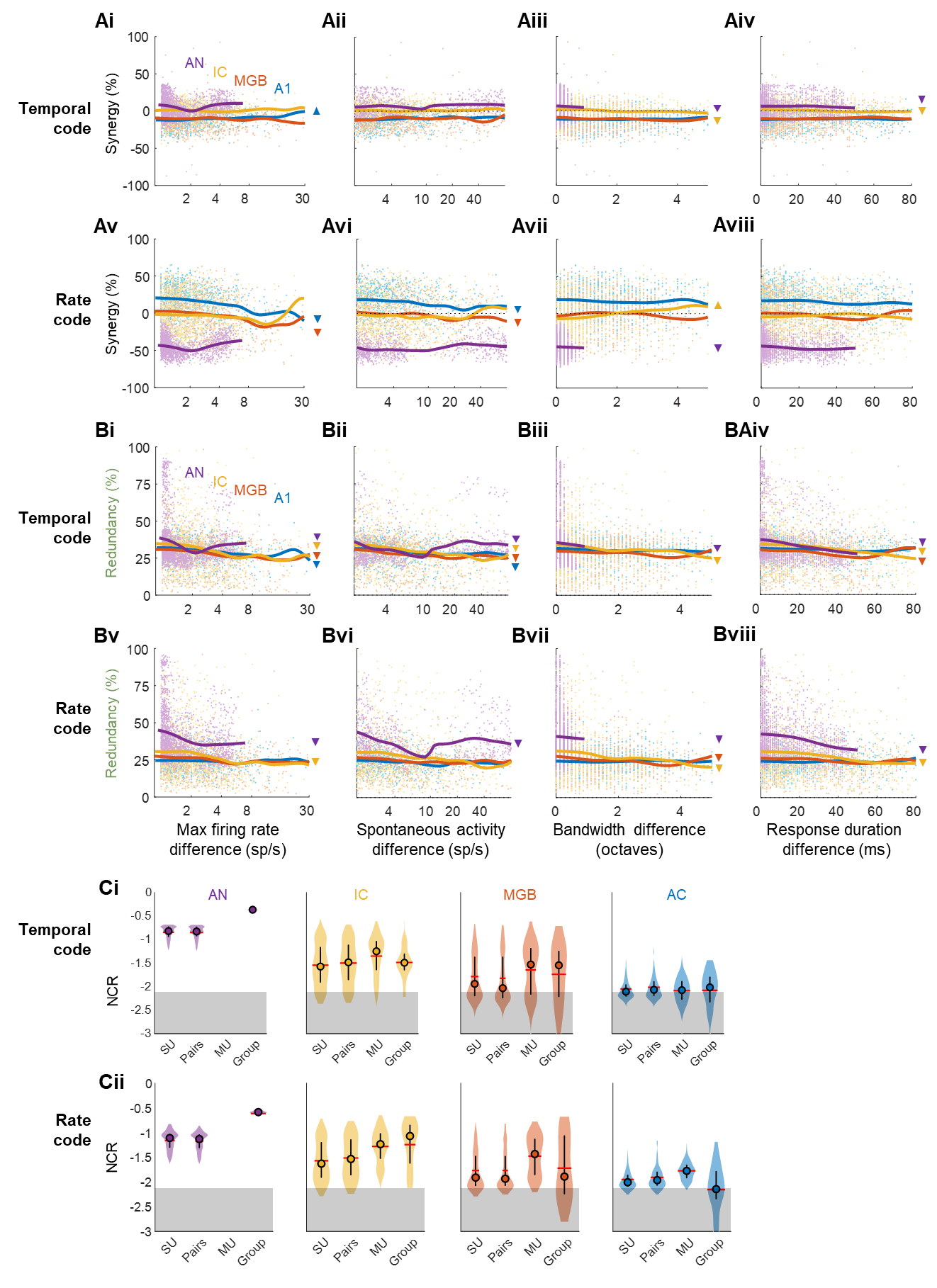


*Supplementary Figure 6 : Complementary analysis of synergy and redundancy to figure 6. A) Synergy for pairs of neurons as a function of the difference between the two neurons in Ai,Av) maximum firing rate, Aii,Avi) spontaneous activity, Aiii,Avii) bandwidth and Aiv,Aviii) peak duration, for temporal (top) and rate (bottom) codes. Solid lines are non-linear regression lines. Significant positive correlations are shown by up-pointing triangles and significant inverse correlations are shown by down-pointing triangles. B) Same as A for redundancy. C) Distribution of NCR values for single units, pairs of neurons, multiunits and set of neurons simultaneously recorded during one electrode penetration (“Group”) for Ci) the rate code and Cii) the temporal code. Ci,Cii) From left to right: data from AN , IC, MGB and A1.*


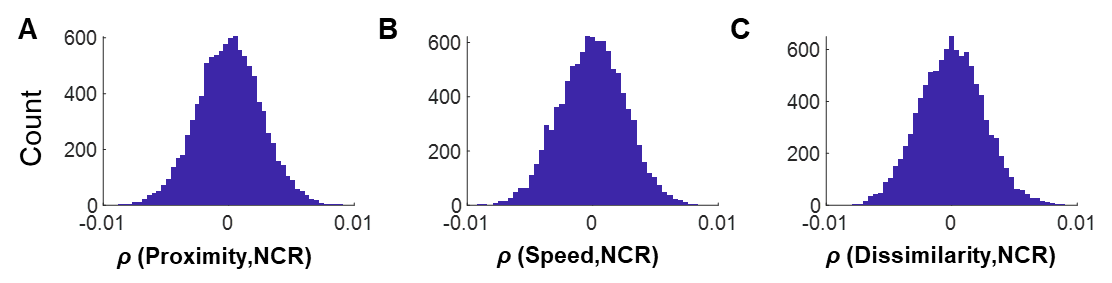


*Supplementary Figure 7 : Distribution of Spearman correlation coefficients between simulated random NCR values and A) proximity, B) average speed and C) dissimilarity values of the RDS stimulus. Percentiles at 2.5 and 97.5% are then taken as significance thresholds for Spearman correlation on real data shown in Figure 7B.*


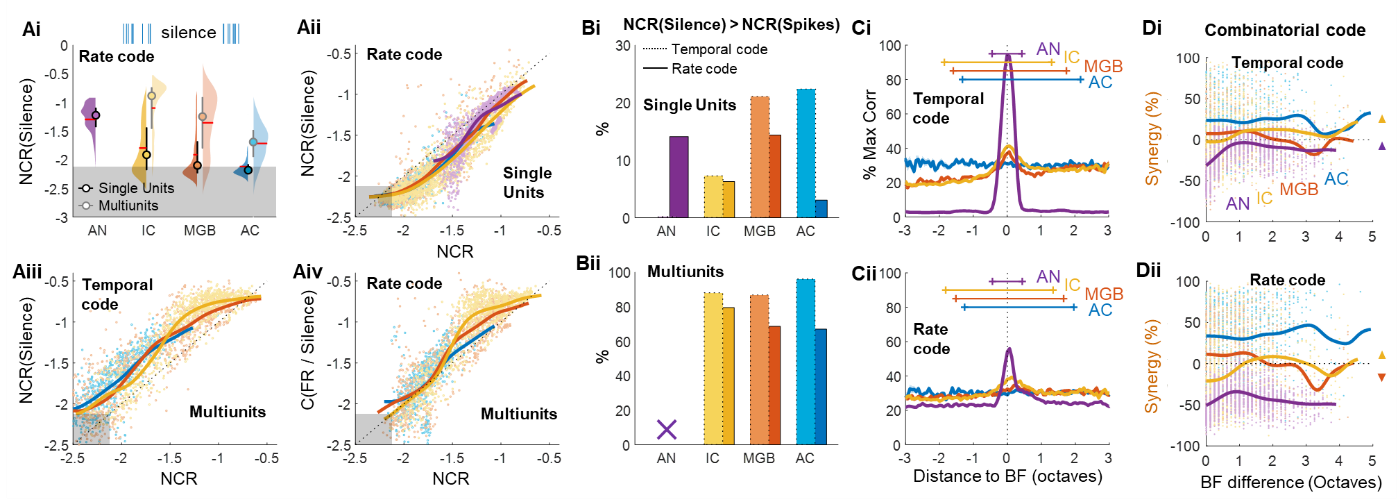


*Supplementary Figure 8 : Ai) NCR for single units (black circles and lines) and multiunits (gray circles and lines) as a function of area for the rate code when considering only periods of neural silence. Aii) NCR for neural silence is plotted against NCR for the whole spike train using the rate code and single units. Aiii) NCR for neural silence is plotted against NCR for the whole spike train using the temporal code and multiunits. Aiv) As in Aiii but considering the rate code. B) Percentage of neurons which show a larger NCR value for silence than NCR values using temporal or rate codes for all studied areas and for Bi) single units and Bii) multiunits. C) Normalized NCR value as a function of the distance to the best frequency (BF) of the neuron for Ci) temporal and Cii) rate codes. Ci,Cii) Only significant NCR values were considered. Average width of STRF significant peaks is shown on the top for each area. D) Synergy for pairs of neurons as a function of BF difference between the two neurons for combinatorial codes associated with Di) temporal and Dii) rate codes. Solid lines are non-linear regression lines. Significant positive correlations are shown by up-pointing triangles and significant inverse correlations are shown by down-pointing triangles.*
